# Supplementary figures and images for: A Drosophila glial cell atlas reveals a mismatch between transcriptional and morphological diversity
Source: PLoS Biol. 2023 Oct 20;21(10):e3002328. doi: 10.1371/journal.pbio.3002328 (PMC10619882; doi:10.1371/journal.pbio.3002328)

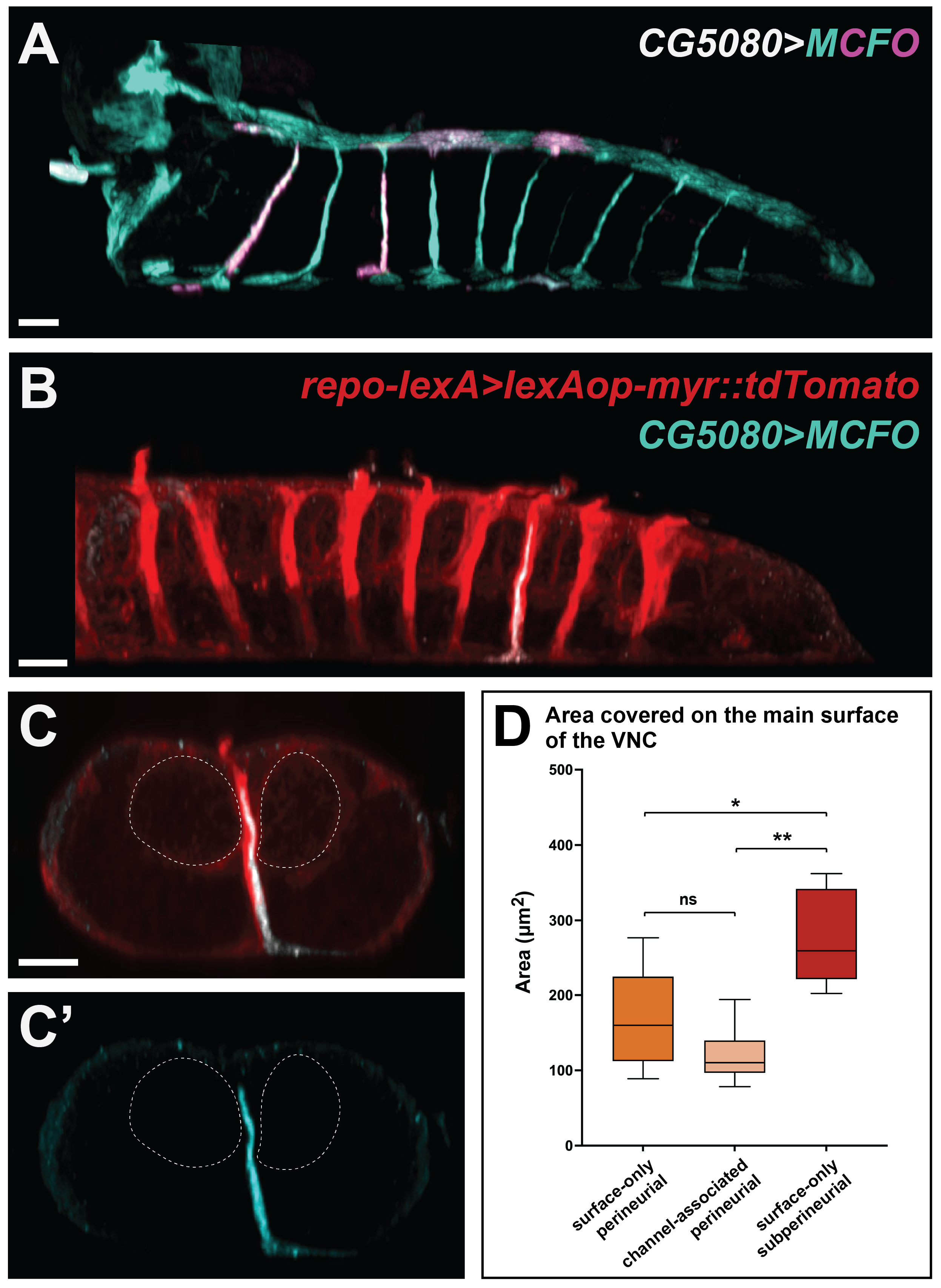

Supplement: S1 Fig — (A) Lateral view of the VNC at 0 h after larval hatching showing channel-associated perineurial glia (cyan and magenta) on the ventral and dorsal surfaces, each sending a single projection with ventral channel-associated perineurial glia sending longer processes than their dorsal counterparts. (B–C’) Lateral view (B) and cross-sectional view (C) of the VNC at 0 h after larval hatching showing a single ventral channel-associated perineurial clone (cyan), and all other glia in red. Note in (B) the red outer glial membranes along the channels that belong to enveloping channel-associated subperineurial glia. Individual channel-associated perineurial glial cells have their surface domains along one side or the other of the midline (C). All scale bars represent 10 μm. (D) Box and whisker plots of the area at the main surface of the VNC occupied by surface-only perineurial glia, channel-associated perineurial glia and surface-only subperineurial glia. N ≥ 6 clones from N ≥ 6 brains. Line in the middle represents the median; box limits represent the 25th and 75th percentiles; whiskers indicate minimum and maximum. Mann–Whitney U-test p-values indicated (ns, non-significant; * p < 0.05; ** p < 0.005). The data underlying this panel can be found in S5 Data. (TIF) [file pbio.3002328.s001.tif]

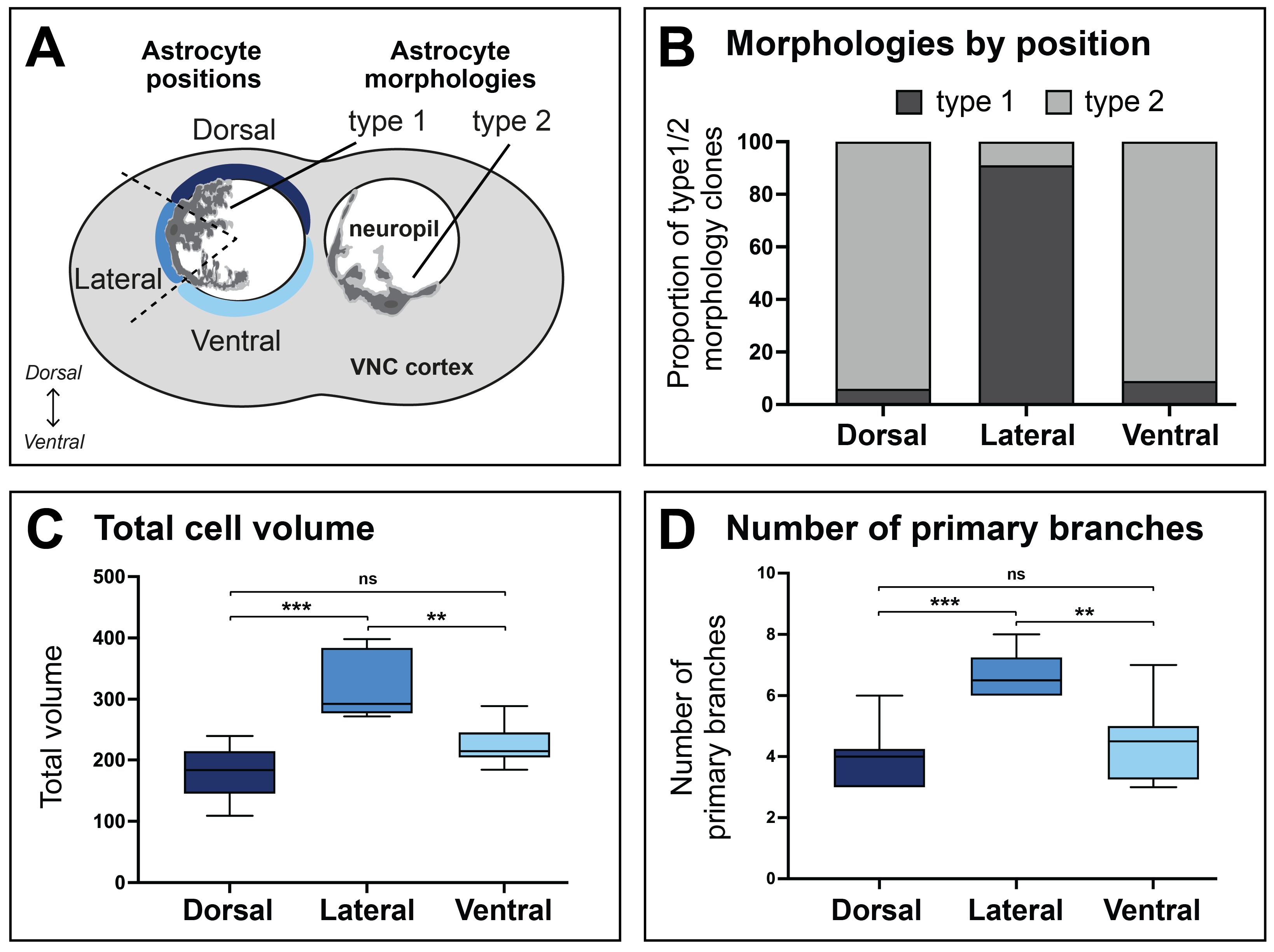

Supplement: S2 Fig — (A) Schematic of the cross-section of the embryonic VNC showing the different astrocyte positions and morphologies. Position types are dorsal, lateral, and ventral based on nucleus position, separated by dashed lines (left). Type 1 morphologies are more arborised and type 2 are less arborised. Morphologies described originate from sparse alrm>MCFO clones. (B) Proportions of type 1 and type 2 morphologies for astrocyte clones at 0 h after larval hatching with nuclei in dorsal, lateral, or ventral positions, as defined in (A). Type 1 astrocytes are more prevalent laterally, while type 2 astrocytes are more prevalent in dorsal and ventral positions. N = 117 clones from N = 36 brains. (C, D) Box and whisker plots of total cell volumes (C) and number of primary branches (D) for astrocyte clones in the dorsal, lateral, and ventral positions. Line in the middle represents the median; box limits represent the 25th and 75th percentiles; whiskers indicate minimum and maximum. The lateral astrocytes, mainly type 1, show higher volumes and more primary branches compared to dorsal and ventral astrocytes, mainly type 2. N ≥ 6 clones from N ≥ 6 brains per neuropil position. Mann–Whitney U-test p-values indicated (ns, non-significant; ** p < 0.01; *** p < 0.001). The data underlying (B–D) can be found in S5 Data. (TIF) [file pbio.3002328.s002.tif]

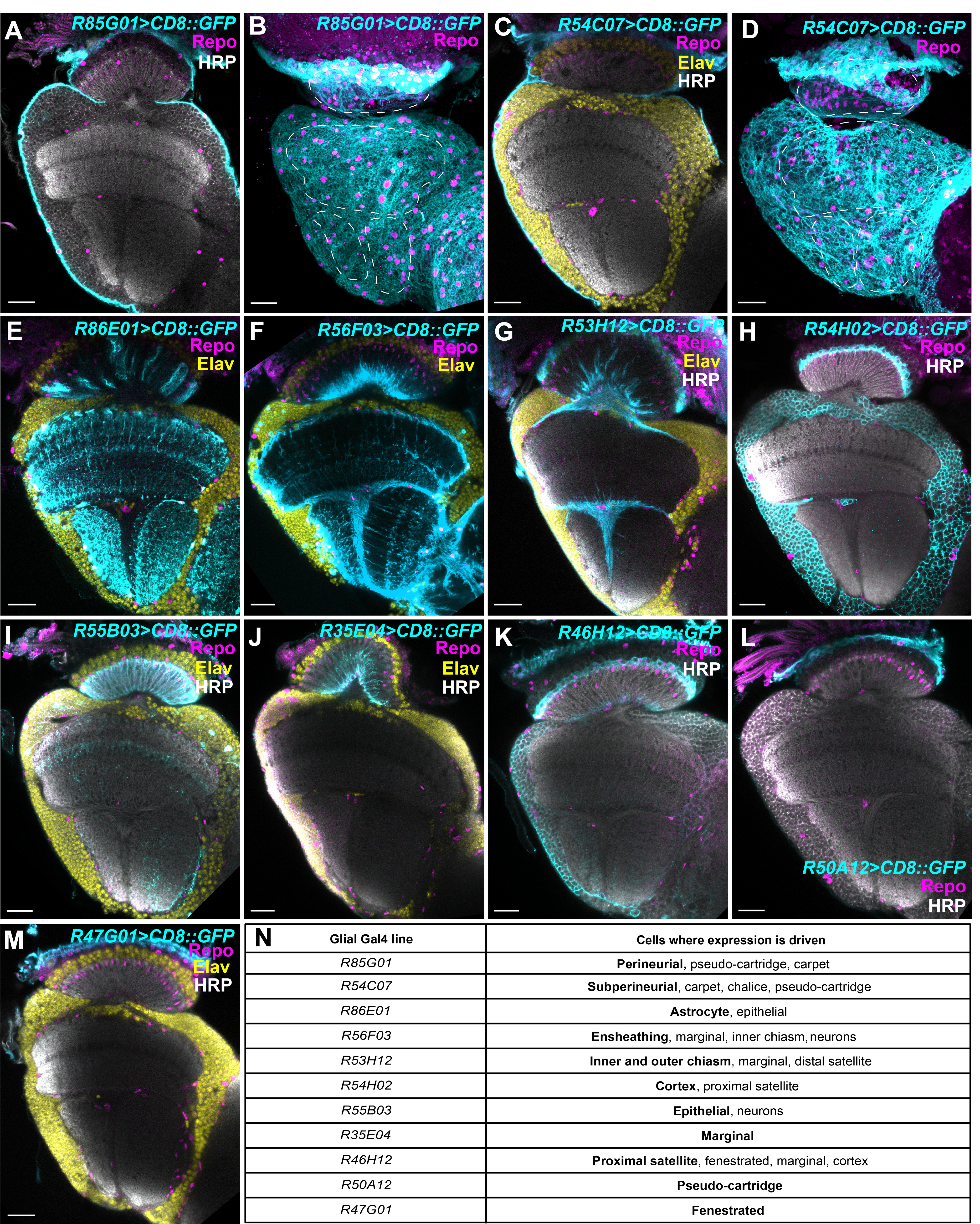

Supplement: S3 Fig — (A–M) GFP expression driven by the indicated glial Gal4 driver (described previously in [25]) for the (A, B) optic lobe perineurial glia, (C, D) optic lobe subperineurial glia, (E) optic lobe astrocyte glia, (F) optic lobe ensheathing glia (not including chiasm glia; also drives expression in a subset of neurons), (G) chiasm glia (and marginal glia), (H) medulla, lobulla, and lobula plate cortex glia and proximal satellite glia, (I) lamina astrocytes (epithelial glia), (J) lamina ensheathing glia (marginal glia), (K) lamina-specific cortex glia (proximal satellite glia), (L) lamina subperineurial glia (pseudo-cartridge glia), and (M) lamina perineurial glia (fenestrated glia). (N) A table outlining the glial Gal4 lines and the glial subtypes that they drive expression within. Where relevant, cyan marks CD8::GFP driven by the glia-Gal4, magenta marks Repo, yellow labels Elav and HRP labels the neuropils in white. Panels B and D are maximum projections showing the surface of the optic lobe. Dashed lines outline the neuropils. All scale bars represent 20 μm. (TIF) [file pbio.3002328.s003.tif]

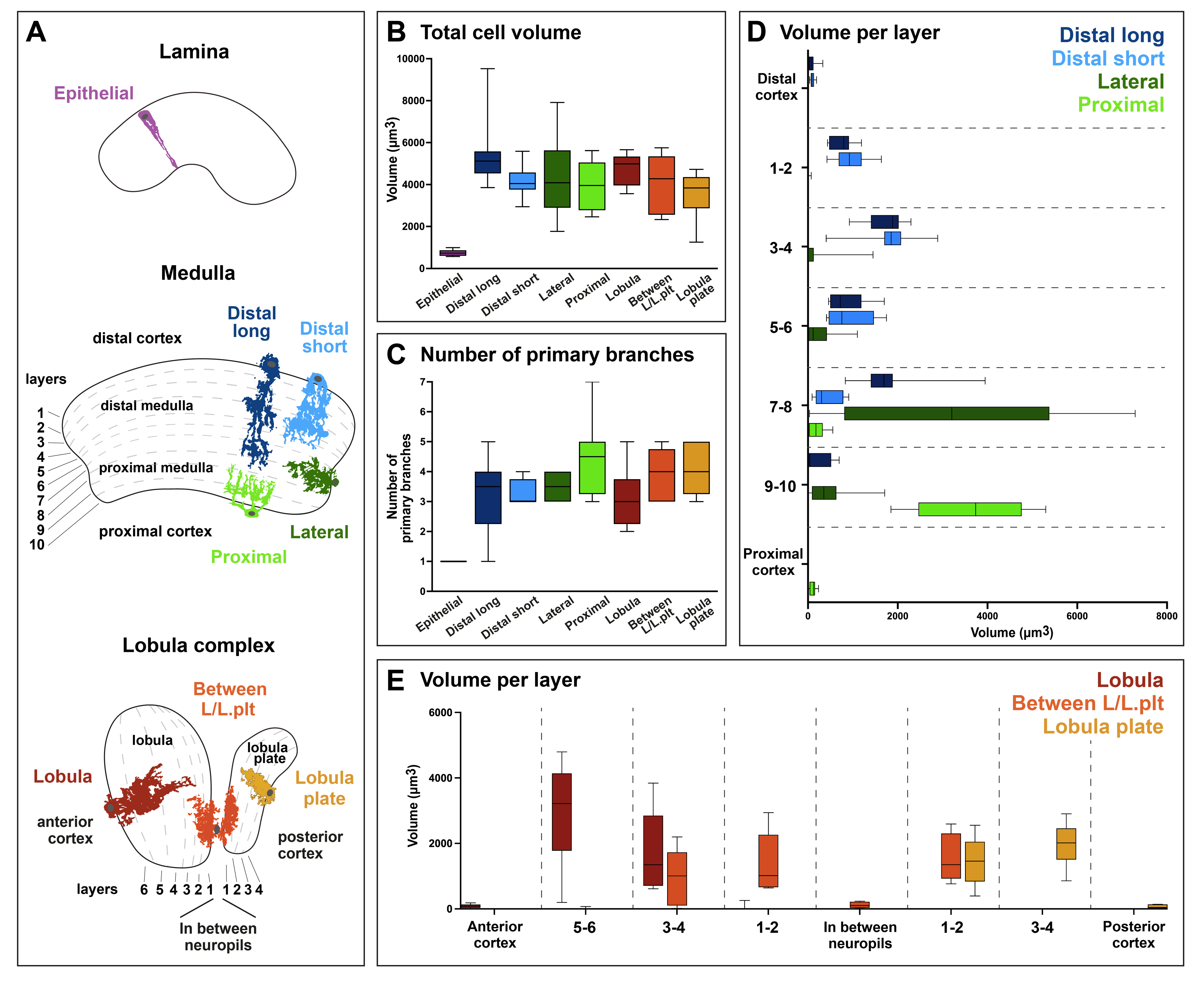

Supplement: S4 Fig — (A) Schematic of the cross-section of the adult optic lobe and its four neuropils: lamina, medulla, lobula, and lobula plate, with the different astrocyte morphologies indicated. Morphologies described originate from sparse Astrocyte(R86E01)>MCFO clones. Dashed lines indicate the neuropil layers. (B, C) Box and whisker plots of total cell volumes (B) and number of primary branches (C) for each astrocyte morphology. N = 8 clones. (D, E) Box and whisker plots of cell volumes within each neuropil layer pair for each astrocyte morphology of the medulla (D) and lobula complex (E). N = 8 clones. Line in the middle represents the median; box limits represent the 25th and 75th percentiles; whiskers indicate minimum and maximum. The data underlying (B–E) can be found in S5 Data. (TIF) [file pbio.3002328.s004.tif]

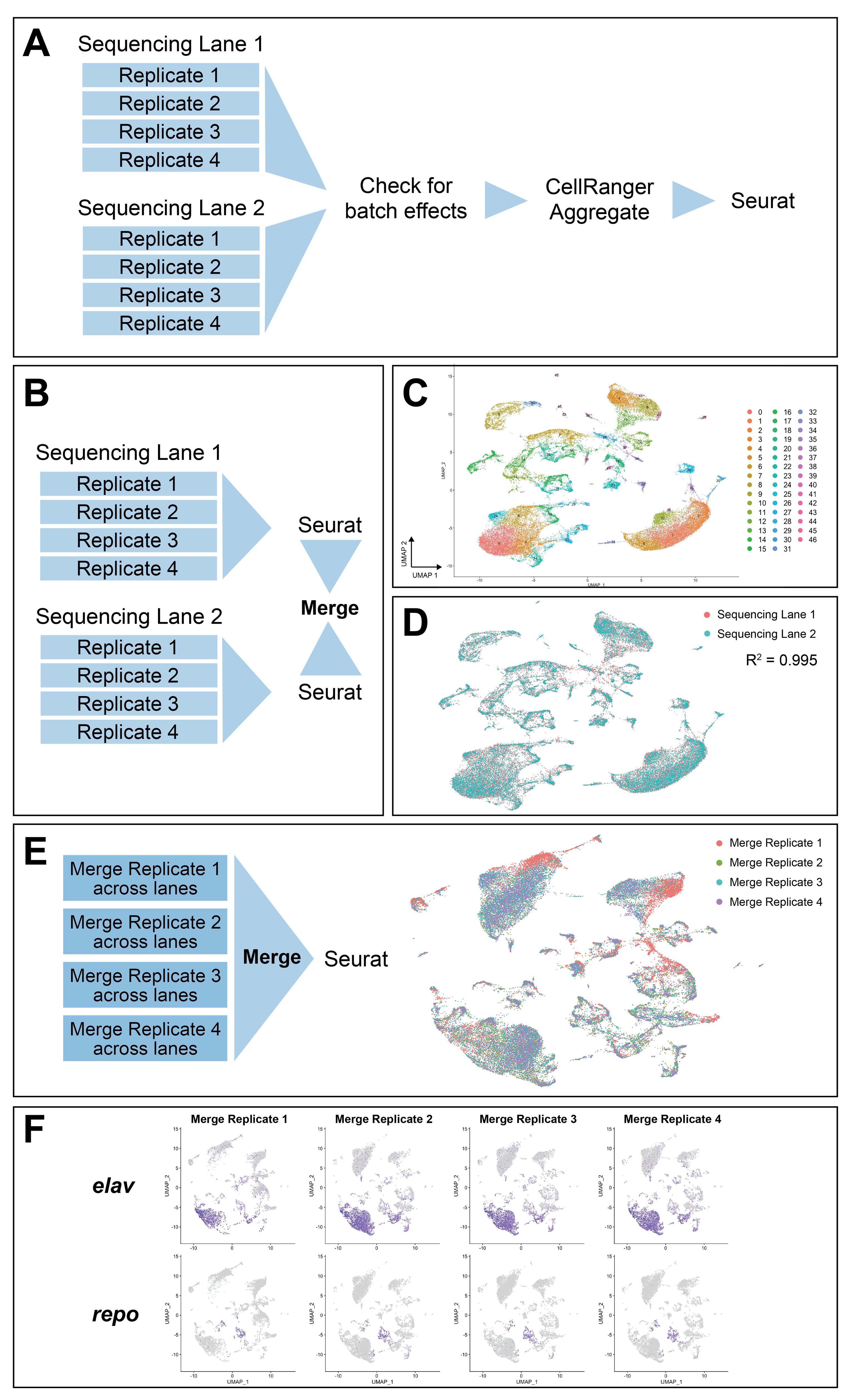

Supplement: S5 Fig — (A) Schematic of data analysis for stage 17 whole embryo; 10X libraries were prepared from 4 biological replicates, each sequenced across 2 separate lanes. Following examination for batch effects (B–F) using the 8 sets of resulting CellRanger outputs, CellRanger Aggregate was used to merge the data. The resulting CellRanger outputs (Sarah_aggr_trial1_barcodes.tsv, Sarah_aggr_trial1_features.tsv, Sarah_aggr_trial1_matrix.mtx) were then read into Seurat for downstream processing (see Materials and methods). (B) Schematic of data analysis to check for batch effects between sequencing lanes. As this analysis predates Seurat integration functionalities, the stage 17 embryo data generated from sequencing lane 1 (biological replicates 1–4) and sequencing lane 2 (biological replicates 1–4) were combined into 2 Seurat objects, merged and clustered (C). The number of cells derived from each sequencing lane were calculated for each cluster in UMAP space, and the correlation between the 2 sequencing lanes was calculated (R2 = 0.995) (see “220706 glia paper supp fig 1.Rmd”). (D) Lane-of-origin was plotted onto the clusters in UMAP space, showing an even distribution across all clusters. Together, these analyses demonstrate the absence of batch effects between sequencing lanes. (E) Schematic of data analysis for examining batch effects between biological replicates. Reads for each biological replicate were combined across both sequencing lanes. These 4 objects were then merged using Seurat and clustered using standard methods. Biological-replicate-of-origin for each cell was plotted onto the clusters in UMAP space. Cells derived from each of the 4 biological replicates contributed equally to the neuronal (illustrated by elav expression) and glial (illustrated by repo expression) clusters of the UMAP (F), demonstrating the absence of batch effects across the replicates. The data underlying this figure can be found at NCBI GEO accession GSE208324 and https://github.com/AustinSeroka [file pbio.3002328.s005.tif]

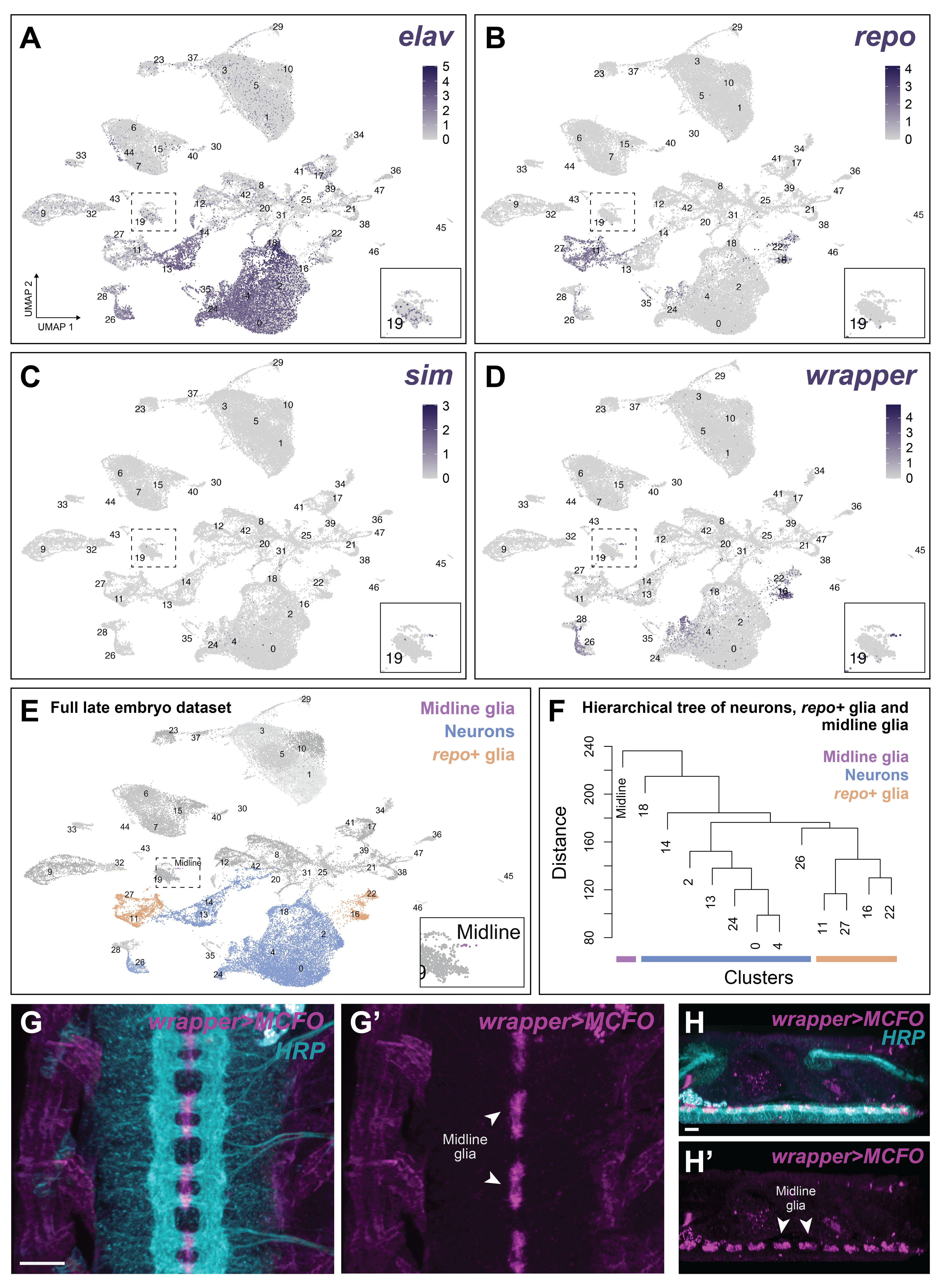

Supplement: S6 Fig — (A–D) Expression levels of elav (A), repo (B), sim (C), and wrapper (D) plotted on the whole embryo UMAP. Each dot represents a single cell, and the colour represents the level of expression as indicated. Zoomed-in details of cluster 19 are shown. (E) UMAP of the whole embryo, indicating clusters defined as midline glia (purple), neurons (blue), and repo+ glia (yellow), based on elav, repo, sim, and wrapper expression. (F) Dendrogram of hierarchical clustering average expression of all genes between midline glia (purple), neurons (blue), and repo+ glia (yellow) clusters. Midline glia form an outgroup to both neuron and repo+ glia. (G, H) Single focal planes of MCFO clones (magenta) of midline glia generated with wrapper-Gal4. HRP in cyan. Scale bar is 10 μm. The data underlying this figure can be found at NCBI GEO accession GSE208324, https://github.com/VilFernandesLab/2022_DrosophilaGlialAtlas and S1 Data. (TIF) [file pbio.3002328.s006.tif]

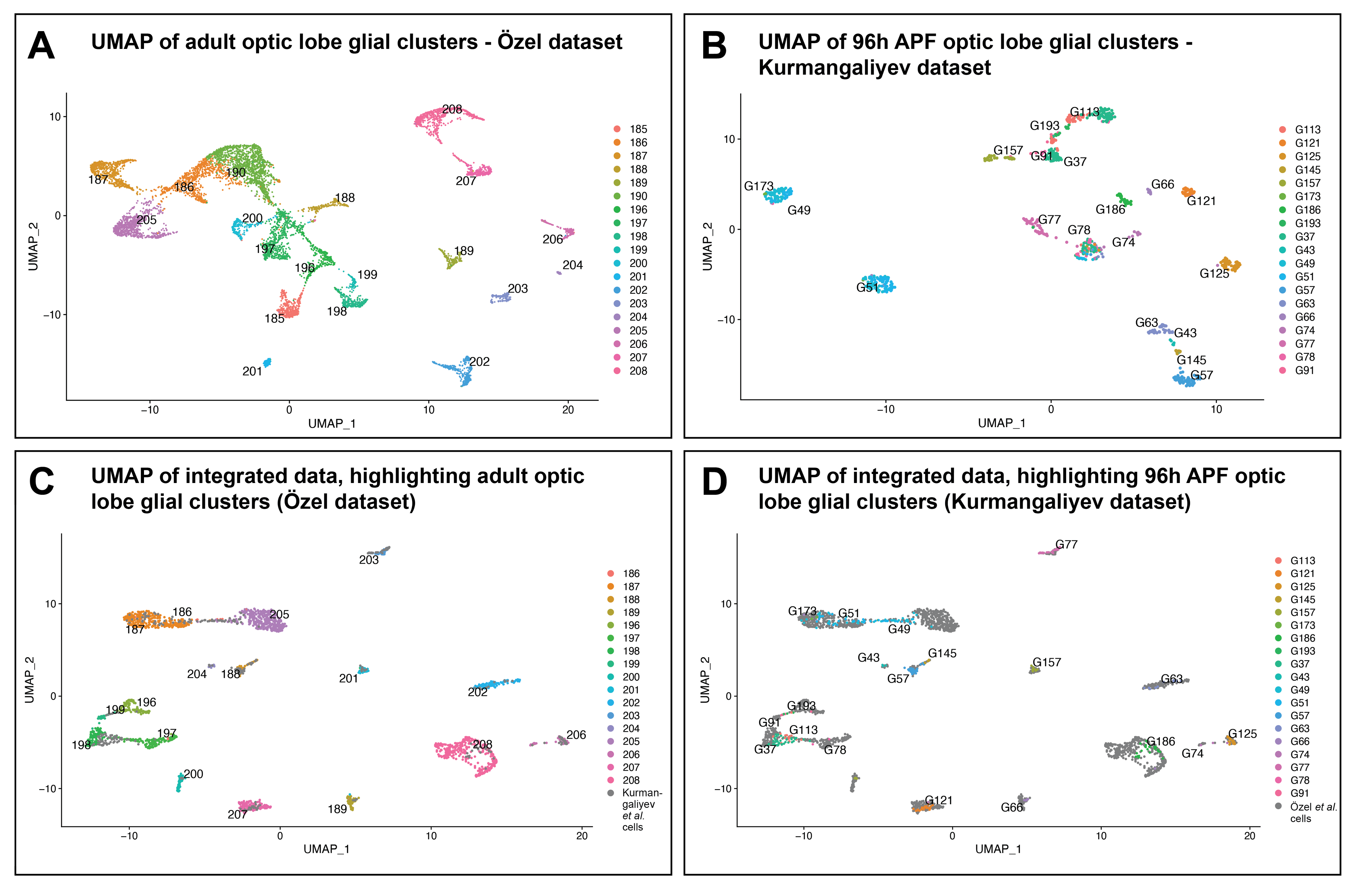

Supplement: S7 Fig — (A) UMAP of the 19 glial clusters from 3-day-old adult optic lobes, from [57]. (B) UMAP of the 19 glial clusters from 96h APF optic lobes, from [58]. (C, D) UMAP of the integrated young adult optic lobe dataset, highlighting the 3-day-old adult clusters (C) or 96h APF clusters (D) in colour, with 96h APF (C) and 3-day-old adult (D) cells in grey. The data underlying this figure can be found at NCBI GEO accessions GSE142787 and GSE156455, and https://github.com/VilFernandesLab/2022_DrosophilaGlialAtlas. (TIF) [file pbio.3002328.s007.tif]

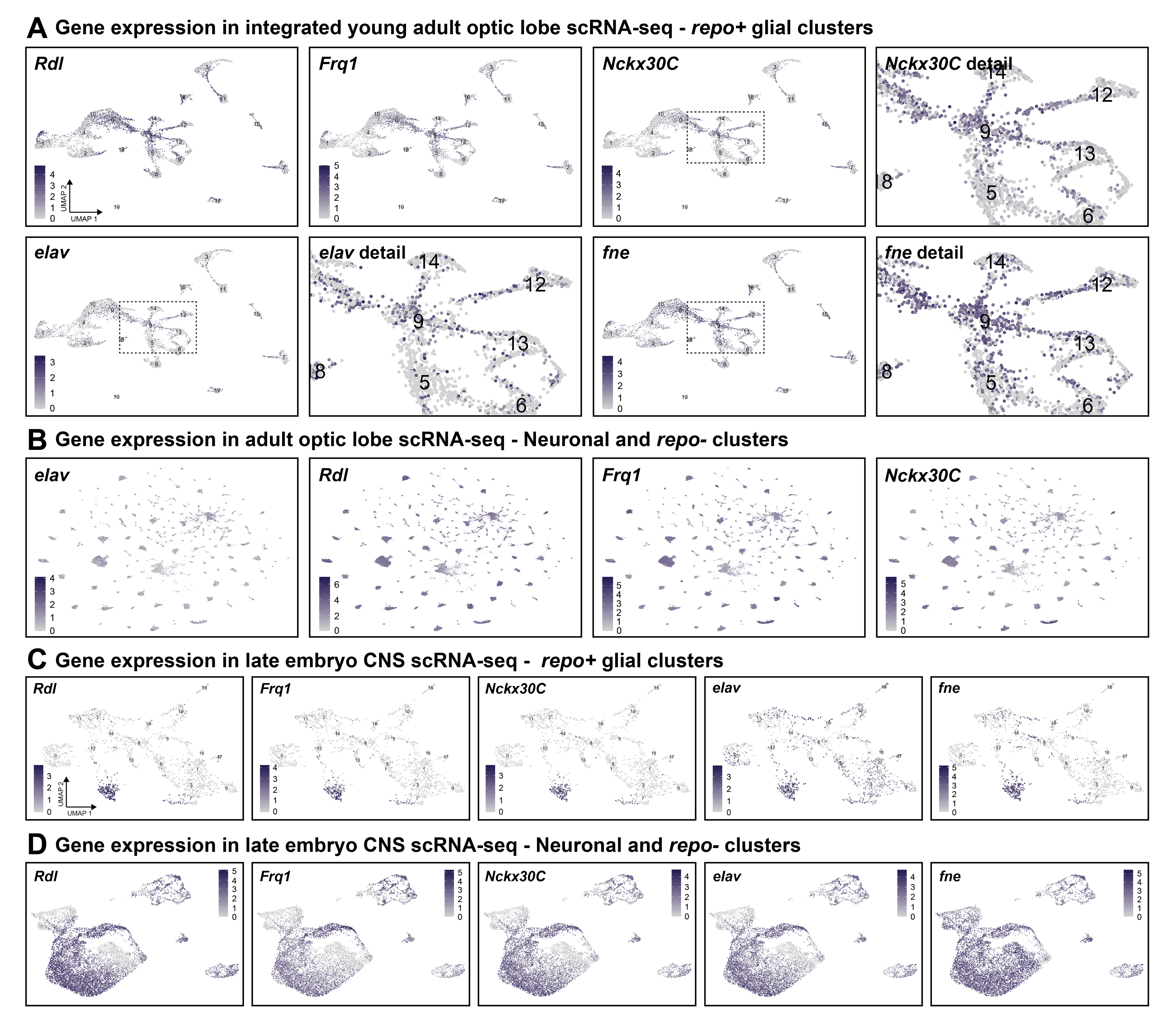

Supplement: S8 Fig — (A) Expression levels of Rdl, Frq1, Nckx30C, elav, and fne plotted on the young adult optic lobe integrated UMAP, before neuronal clean-up. Each dot represents a single cell, and the colour represents the level of expression as indicated. Zoomed-in details of the centre of the UMAP are shown for Nckx30C, elav, and fne. (B) Expression levels of elav, Rdl, Frq1, and Nckx30C plotted on the 3-day-old adult optic lobe UMAP, from [57], including all clusters except the 19 glial clusters. All 4 genes showed expression in all clusters, illustrating the pan-neuronal nature of Rdl, Frq1, and Nckx30C expression. (C) Expression levels of Rdl, Frq1, Nckx30C plotted on the embryonic glial UMAP, before neuronal clean-up. The expression of these 3 genes overlapped with the expression of elav and fne, mainly in cluster #4. (D) Expression levels of Rdl, Frq1 and Nckx30C elav and fne plotted on the UMAP of the embryonic nervous system clusters. The data underlying this figure can be found at NCBI GEO accessions GSE142787 and GSE156455, and https://github.com/VilFernandesLab/2022_DrosophilaGlialAtlas. (TIF) [file pbio.3002328.s008.tif]

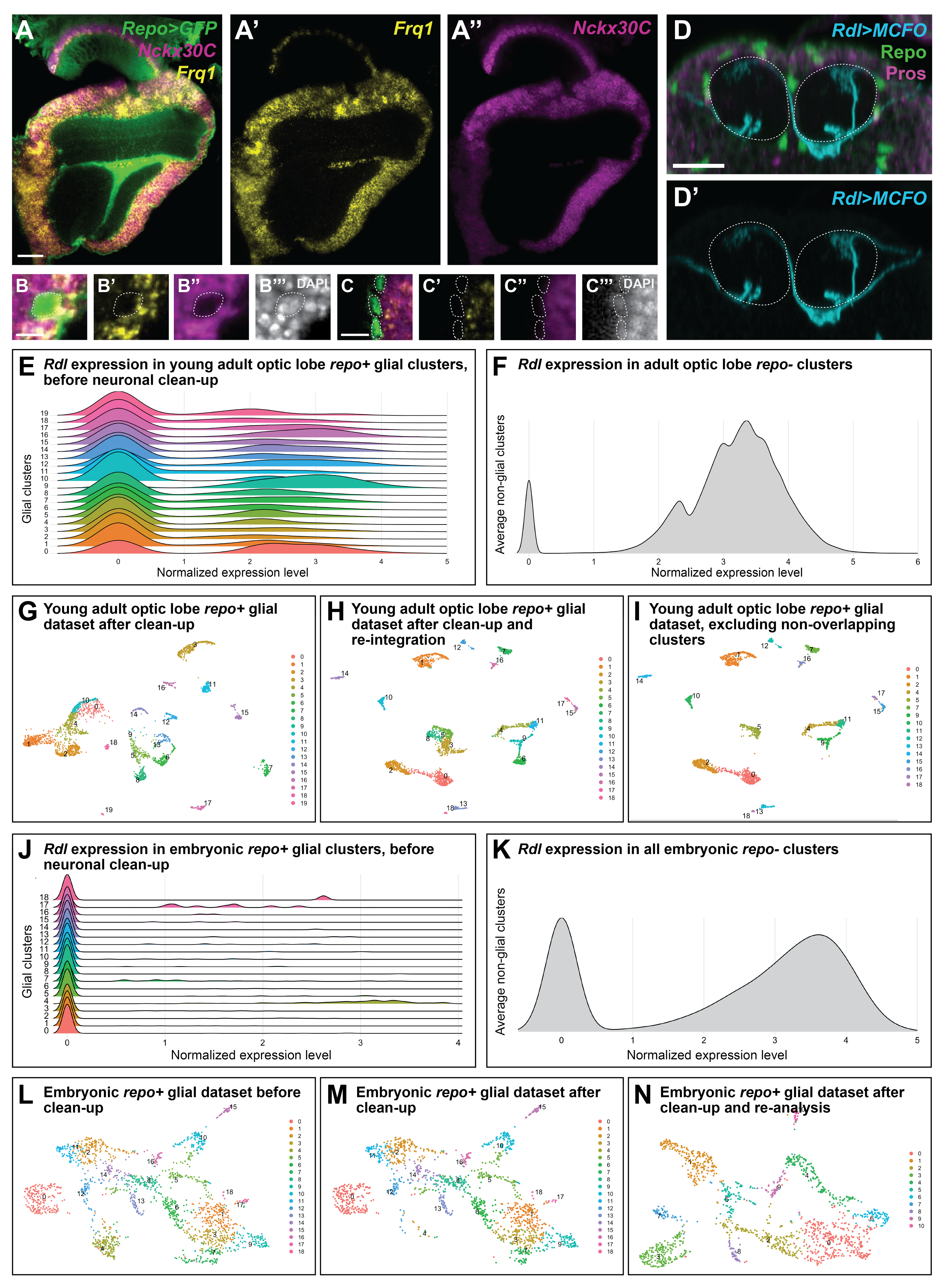

Supplement: S9 Fig — (A) Frq1 (yellow) and Nckx30C (magenta) expression were detected by in situ HCR in the cortex area of the optic lobe. GFP labelled all glial cells in green. Single focal plane. Scale bar is 20 μm. (B, C) Single focal planes showing glial somas (green) with Frq1 (yellow) and Nckx30C (magenta) expression. DAPI marks all nuclei in white. Dashed lines outline glial somas. Scale bars are 5 μm. (D) Single focal planes of MCFO clones (cyan) generated with Rdl-Gal4. Repo in green and Prospero in magenta. Dashed lines outline the neuropil. Scale bar is 7 μm. (E) The distribution of Rdl expression levels in each glial cluster of the young adult optic lobe integrated dataset, before neuronal clean-up. (F) The distribution of Rdl expression levels in all cells of the 3-day-old adult optic lobe UMAP, from [57], except glial clusters. (G) UMAP of the 20 glial clusters obtained from the first integration of optic lobe datasets. (H) UMAP of 19 glial clusters after clean-up of potential neurons by excluding cells with normalised expression of Rdl, Frq1, and Nckx30C >1, and cells with Hml expression (>0 normalised expression) as potential hemocytes. (I) Cluster #3, #6, and #8 were excluded from the UMAP in (H) since less than 1% of the cells contained in them originated from the Kurmangaliyev dataset (see Materials and methods for details). (J) The distribution of Rdl expression in each glial cluster of the embryonic dataset, before neuronal clean-up. (K) The distribution of Rdl expression in all cells of the embryonic nervous system, excluding cells belonging to the 19 glial clusters. (L) UMAP of the 19 initial glial clusters of the embryonic nervous system, before neuronal clean-up. (M) Same UMAP as in (L) after exclusion of potential neurons (normalised expression >1 of Rdl, Frq1, and Nckx30C) and potential hemocytes (normalised expression >0 of Hml expression). (N) UMAP of the remaining cells in (M) after reanalysis and reclustering (see Materials and methods for details). The da [file pbio.3002328.s009.tif]

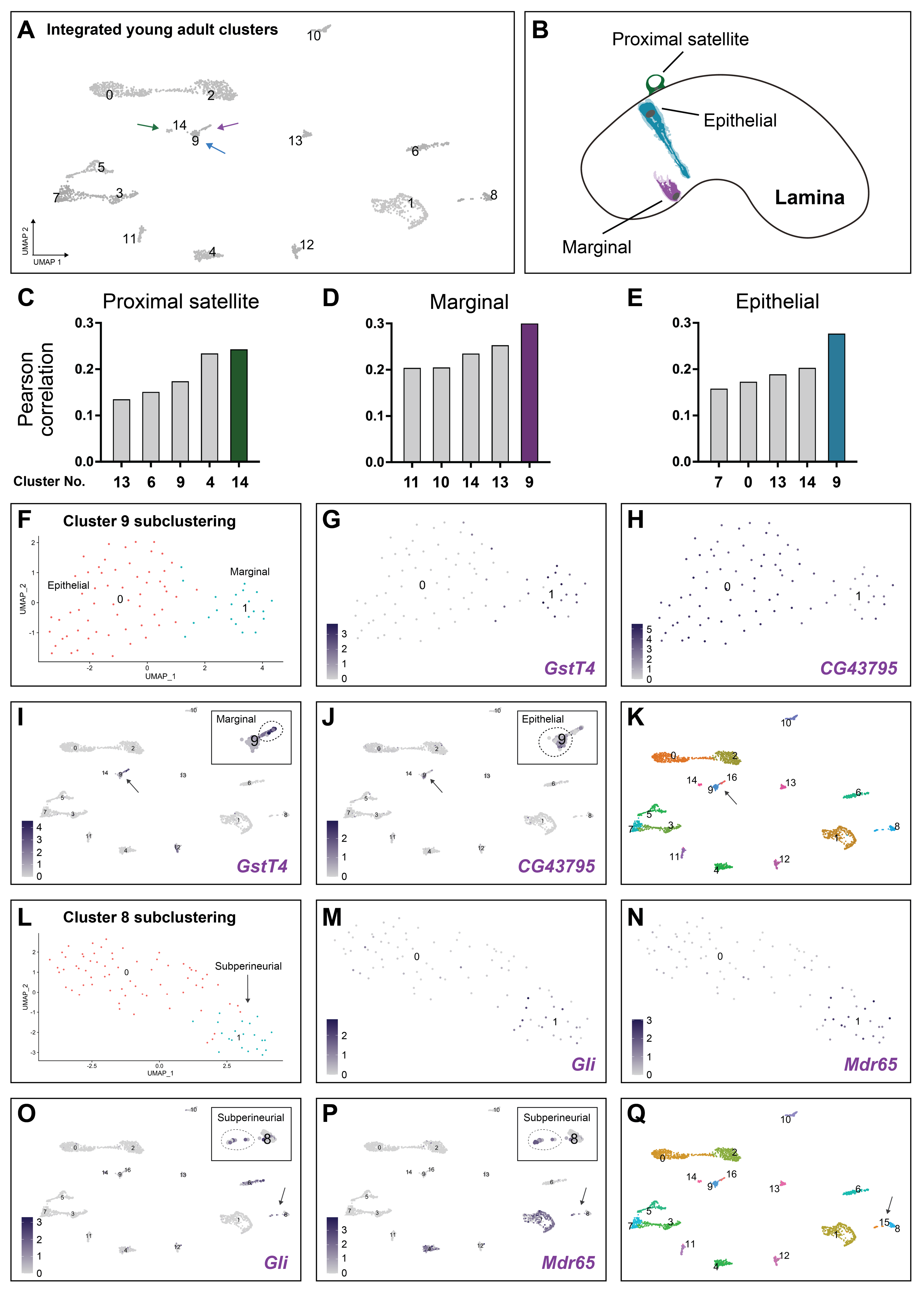

Supplement: S10 Fig — (A) UMAP of the 15 glial clusters of the integrated young adult dataset. (B) Schematic of the adult optic lobe lamina with marginal, epithelial, and proximal satellite glia represented. (C–E) Pearson correlation values between the bulk-RNA-seq transcriptomes of the proximal satellite, marginal and epithelial glia and the top 5 glial cluster matches from the young adult dataset. (F) Subclustering of cluster #9 generated 2 subclusters. (G) GstT4 (a gene highly expressed marginal glia bulk RNA-seq data) showed high expression in subcluster #1. (H) CG43795 (a gene highly expressed in epithelial glia bulk RNA-seq) showed high expression in subcluster #0. (I, J) Expression of GstT4 (I) and CG43795 (J) in the UMAP of the 15 glial clusters of the integrated young adult dataset. Both genes showed mutually exclusive expression patterns within cluster #9. (K) UMAP showing the original cluster #9 split into new clusters #9 and #16 (see Materials and methods). (L) Subclustering cluster #8 generated 2 subclusters. (M, N) Gli (Gliotactin) and Mdr65 (Multi drug resistance 65), both known markers of subperineurial glia [105], were expressed in subcluster #1. (O, P) Expression of Gli and Mdr65 on the UMAP of the 16 glial clusters of the integrated young adult dataset. Both genes showed overlapped expression in a group of cells belonging to cluster #8. (Q) UMAP showing the original cluster #8 split into new clusters #8 and #15 (see Materials and methods). The data underlying this figure can be found in S3, S5, and S7 Data files. (TIF) [file pbio.3002328.s010.tif]

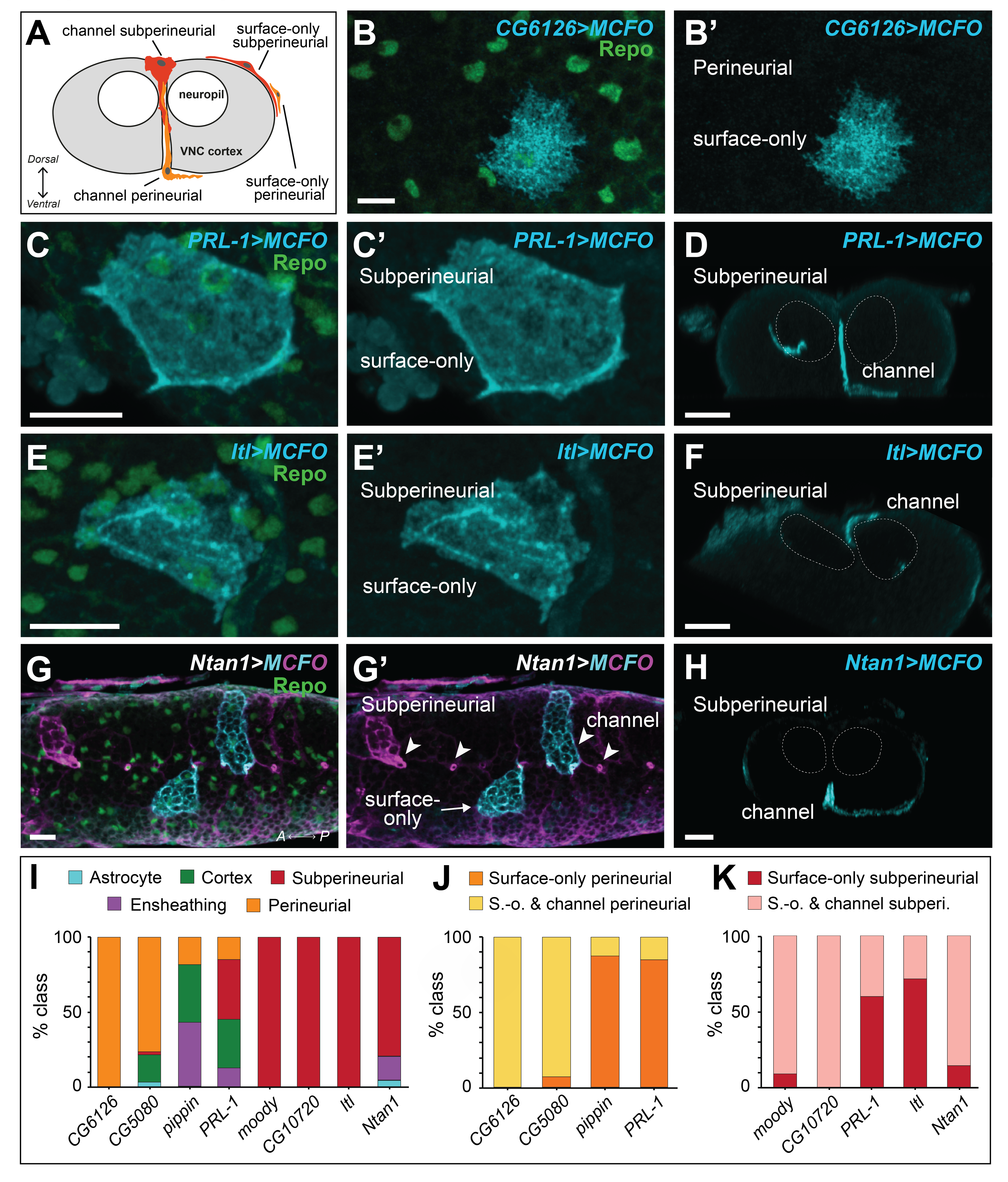

Supplement: S11 Fig — (A) Schematic of the cross-section of the embryonic VNC showing the different surface glial types, channel-associated and surface-only perineurial or subperineural glia. (B–H) Surface and cross-sectional views of MCFO clones at 0 h after larval hatching generated with the Gal4 lines indicated, belonging to marker genes with high expression in the surface glia clusters: CG6126 (N = 372 clones from N = 11 brains), PRL-1 (N = 154 clones from N = 17 brains), ltl (N = 19 clones from N = 7 brains), and Ntan1 (N = 68 clones from N = 14 brains). All MCFO clones labelled in cyan and magenta, with Repo in green. (I) Quantification of the frequency of clones recovered by glial class for the indicated driver. (J) Quantification of perineurial glia morphotype frequency by driver line. Channel-associated perineurial glia were detected in 100% of CG6126 MCFO brains (N = 11 brains total) and 92.9% of CG5080 brains (N = 13 brains total), compared to 7.7% of pippin MCFO brains (N = 13 brains total) and 5.7% of PRL-1 MCFO brains (N = 15 brains total). (K) Quantification of subperineurial glia morphotype frequency by driver line. Channel-associated subperineurial glia were detected in 90.9% of moody MCFO brains (N = 12 brains total), 28.6% of ltl MCFO brains (N = 7 brains total), 85.7% of Ntan1 MCFO brains (N = 15 brains total), 100% of CG10702 MCFO brains (N = 15 brains total), and 40% of PRL MCFO brains (N = 15 brains total). Dashed lines outline the neuropil. All scale bars are 10 μm. The data underlying (I–K) can be found in S5 Data. (TIF) [file pbio.3002328.s011.tif]

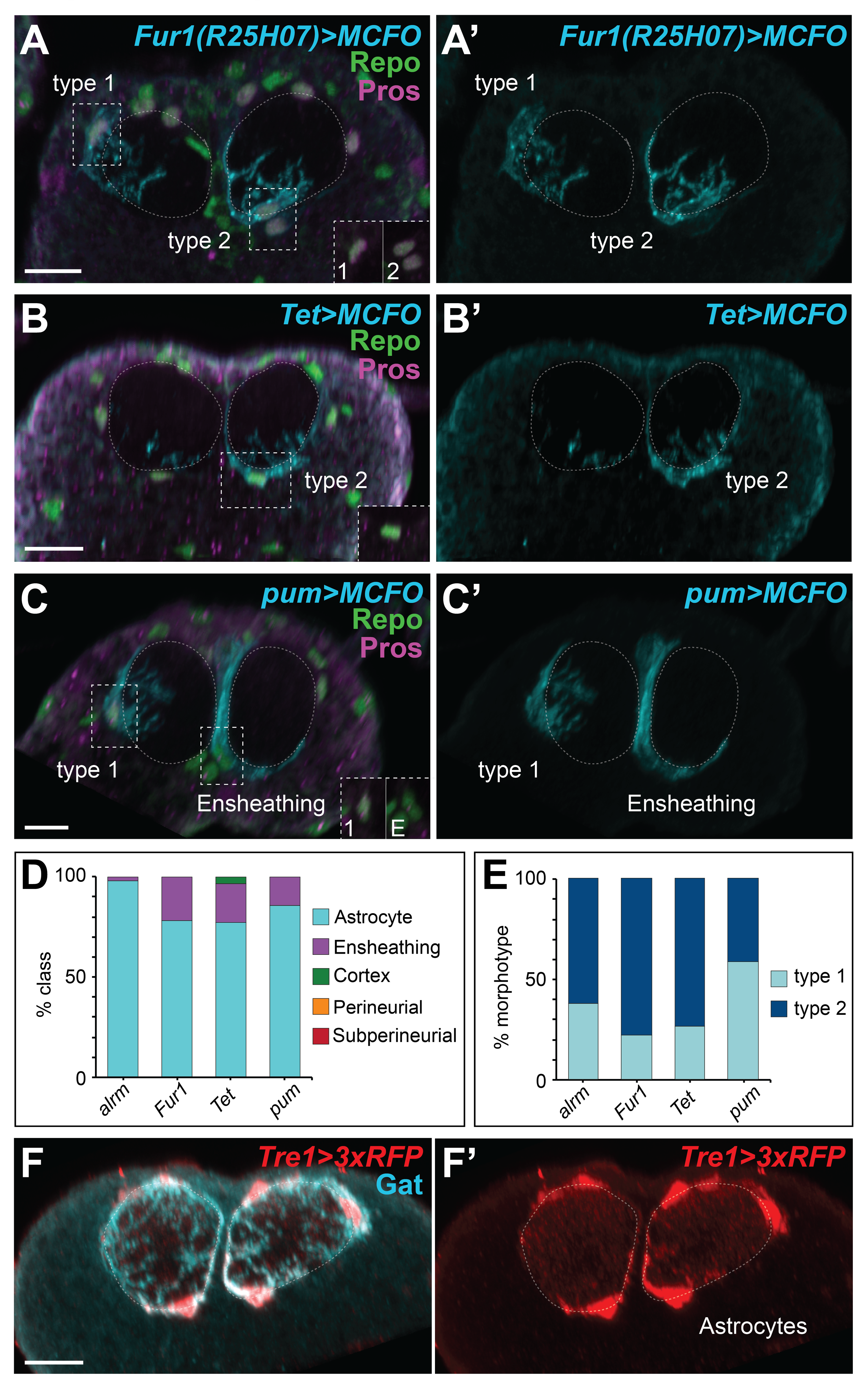

Supplement: S12 Fig — (A–C) MCFO clones (VNC cross sections) at 0 h after larval hatching generated with the Gal4 lines indicated, belonging to marker genes with high expression in the astrocyte cluster: Fur1 (N = 27 clones from N = 9 brains), Tet (N = 71 clones from N = 10 brains), and pum (N = 25 clones from N = 9 brains). All MCFO clones labelled in cyan, with Repo in green and Prospero in magenta. Insets in (A–C) show Prospero and Repo in glial nuclei; only astrocyte clones were positive for Prospero. Dashed lines outline the neuropils. (D) Quantification of the frequency of glial type clone for each indicated driver line: alrm (N = 117 clones from N = 36 brains), other Ns noted above. (E) Quantification of astrocyte morphotype frequency by driver line. (F) VNC cross section showing colocalization of the astrocyte marker Gat (cyan) and a gene-trap line where 3xRFP has been swapped for the Tre1 locus (red). Dashed lines outline the neuropil. Scale bars are 10 μm. The data underlying (D, E) can be found in S5 Data. (TIF) [file pbio.3002328.s012.tif]

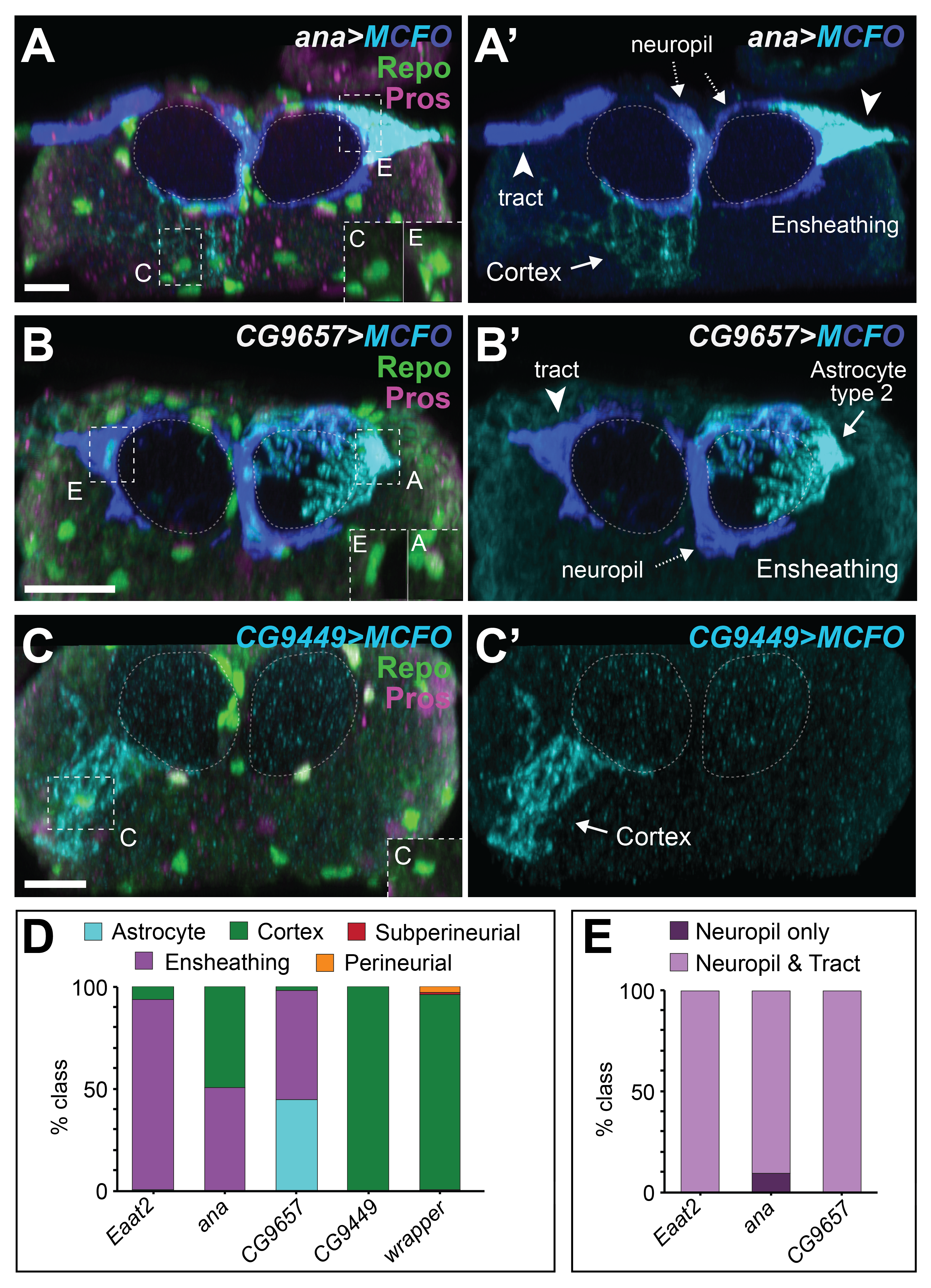

Supplement: S13 Fig — (A–C) MCFO clones (VNC cross sections) at 0 h after larval hatching generated with the Gal4 lines indicated, belonging to marker genes with high expression in the ensheathing or cortex clusters: ana (N = 388 clones from N = 11 brains), CG9657 (N = 287 clones from N = 10 brains), and CG9449 (N = 5 clones from N = 3 brains). Tract and neuropil ensheathing types are indicated. All MCFO clones labelled in cyan and blue, with Repo in green and Prospero in magenta. Insets in (A, B, C) show Prospero and Repo in glial nuclei, where only astrocyte clones were positive for Prospero. Dashed lines outline the neuropils. (D) Quantification of the frequency of glial type clone for each indicated driver line: wrapper (N = 13 brains), other Ns noted above. (E) Quantification of the frequency of brains with clones of both ensheathing types, tract and neuropil, or only neuropil clones, for each indicated driver line. Dashed lines outline the neuropil. Scale bars are 10 μm. The data underlying (D, E) can be found in S5 Data. (TIF) [file pbio.3002328.s013.tif]

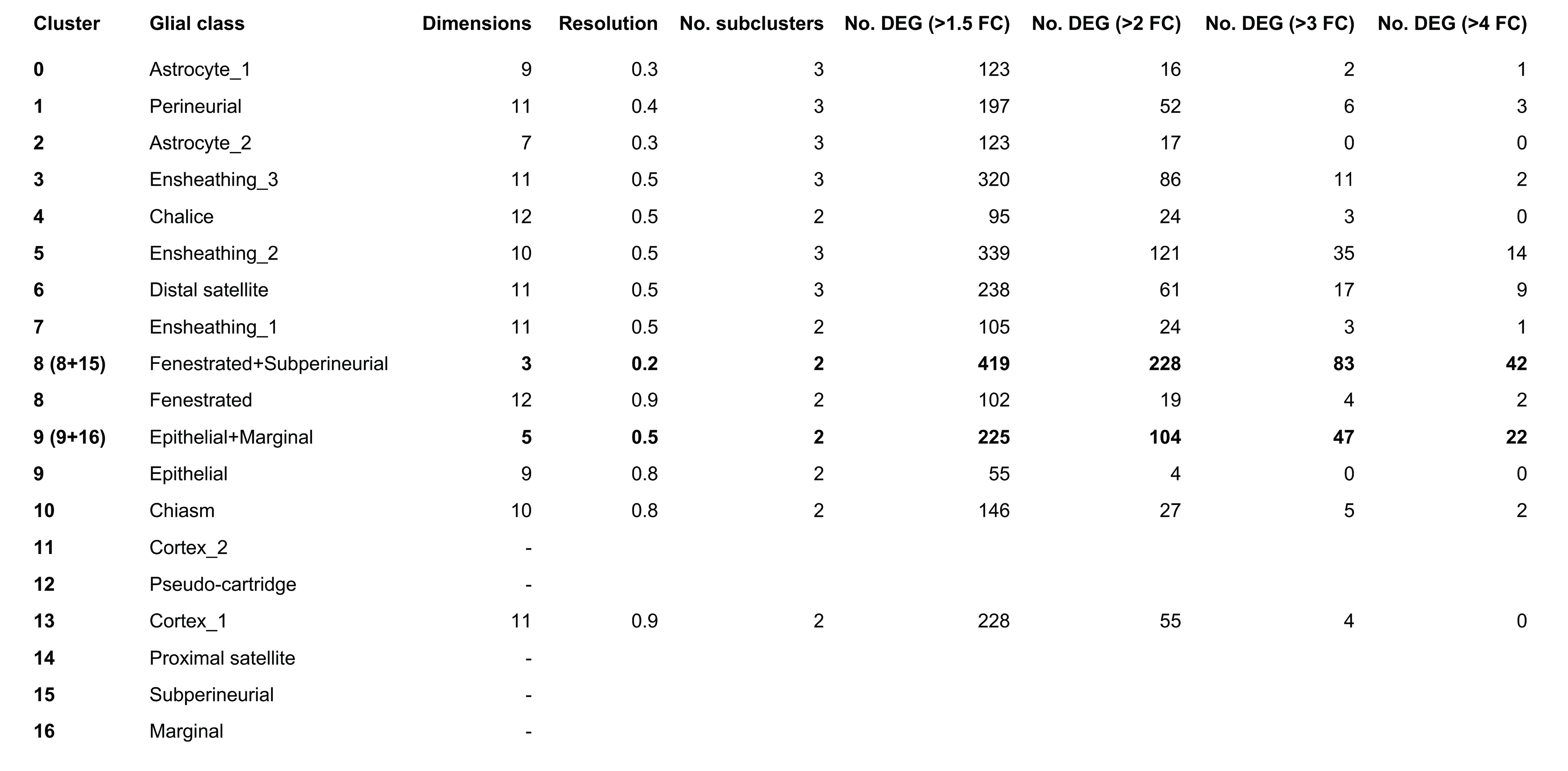

Supplement: S14 Fig — The data underlying this figure can be found in S7 Data and https://github.com/VilFernandesLab/2022_DrosophilaGlialAtlas. (TIF) [file pbio.3002328.s014.tif]

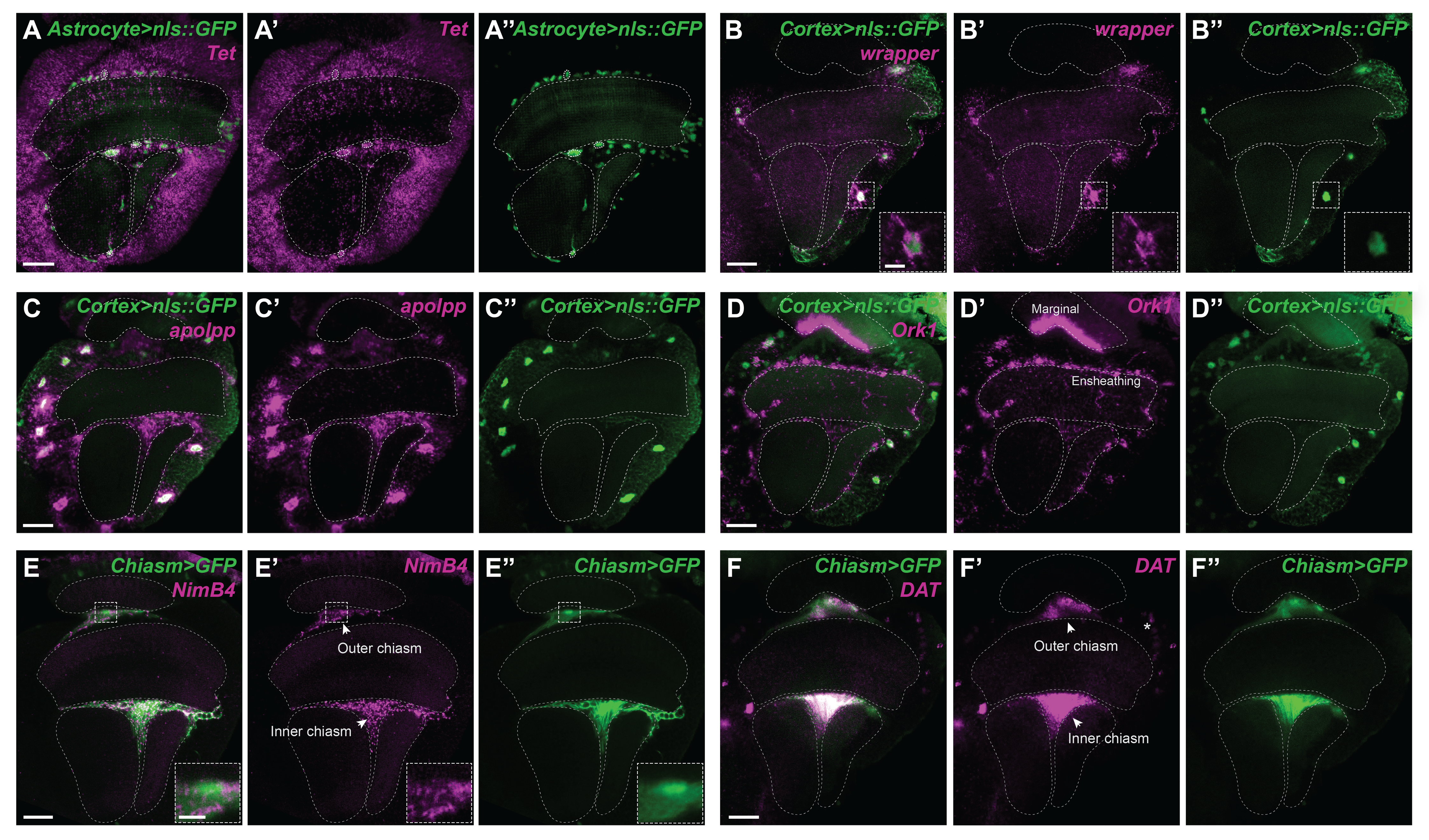

Supplement: S15 Fig — (A) Astrocyte(R86E01)>nls::GFP adult optic lobe where Tet (Ten-Eleven Translocation (TET) family protein) expression (magenta) was detected by in situ HCR throughout the cortex, including in many GFP positive astrocyte (green) nuclei (outlined by thicker dashed lines). See Figs 5 and 6 for additional in vivo astrocyte marker gene validation. (B) Cortex(R54H02)>nls::GFP adult optic lobe where wrapper expression (magenta) was detected by in situ HCR in many GFP positive cortex glia (green) nuclei. Inset of zoomed in nuclei (scale bar is 5 μm). (C) Cortex(R54H02)>nls::GFP adult optic lobe where apolpp (apolipophorin) expression (magenta) was detected by in situ HCR in most GFP positive (green) nuclei. A few nuclei adjacent to the neuropil were also positive (likely ensheathing glia based on the scRNA-seq data). (D) Cortex(R54H02)>nls::GFP adult optic lobe where Ork1 (Open rectifier K+ channel 1) expression (magenta) was detected by in situ HCR in some GFP positive cortex glia (green) nuclei. Many nuclei adjacent to the neuropil were also positive (likely ensheathing glia based on the scRNA-seq data), as well as in marginal glia. (E) Chiasm(R53H12)>GFP adult optic lobe where NimB4 (Nimrod B4) expression (magenta) was detected by in situ HCR in most GFP positive chiasm glia (green) nuclei. Inset of zoomed in nuclei (scale bar is 5 μm). (F) Chiasm(R53H12)>GFP adult optic lobe where DAT (Dopamine transporter) expression (magenta) was detected by in situ HCR in most GFP positive chiasm glia (green) nuclei. Some nuclei in the cortex region around the medulla were positive for DAT (asterisk), consistent neuronal expression in the scRNA-seq data from [57]. Single focal planes in (A, E, F) and maximum projections of 11–12 focal planes (1 μm each) in (B–D). Dashed lines outline the neuropils and scale bars are 20 μm. (TIF) [file pbio.3002328.s015.tif]

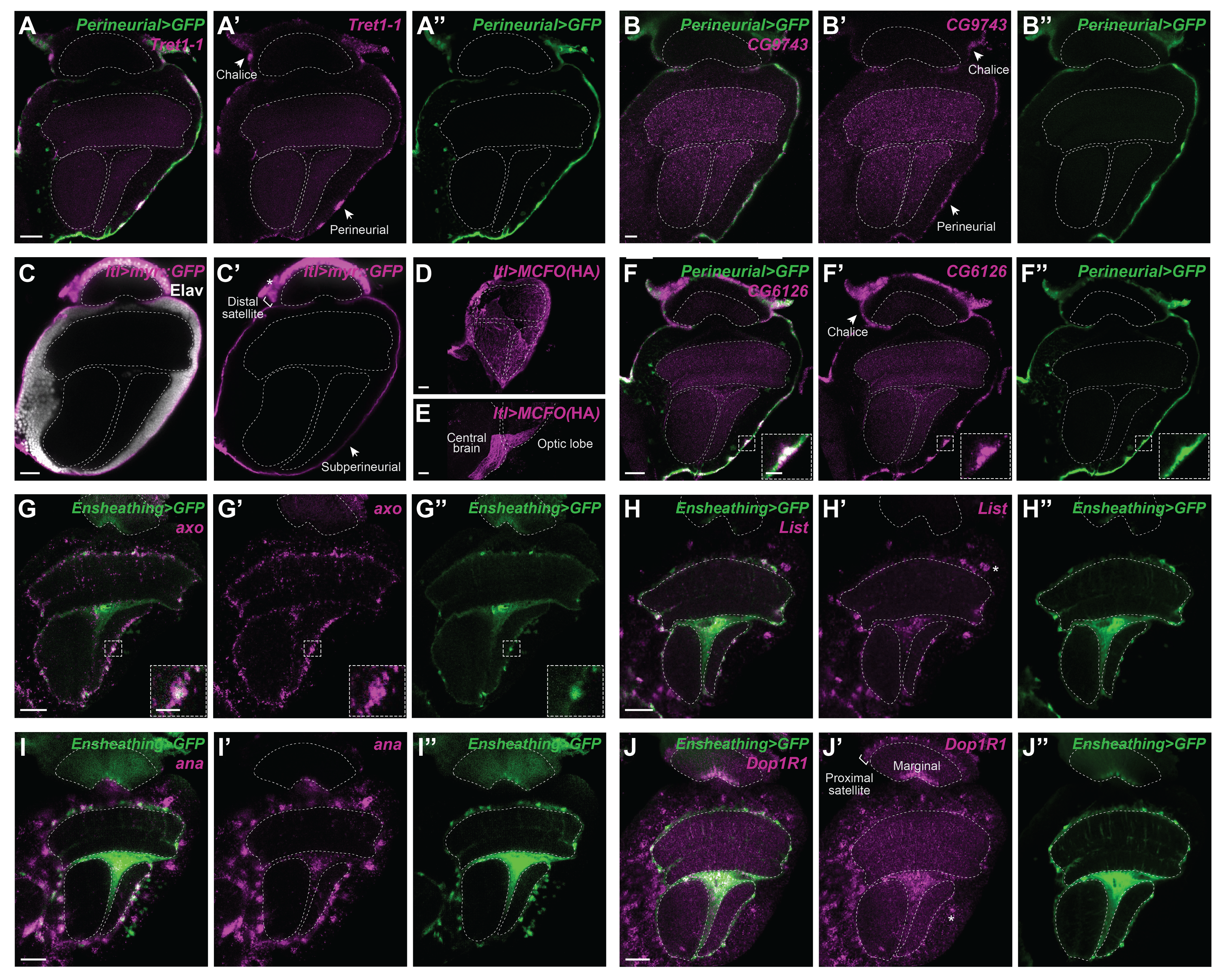

Supplement: S16 Fig — (A, B) Perineurial(R85G01)>GFP adult optic lobe where (A) Tret1-1 (Trehalose transporter 1–1) and (B) CG9743 expression (magenta) was detected by in situ HCR. For both genes, most GFP positive (green) nuclei of the general perineurial glia (surrounding the medulla, lobula, and lobula plate) were positive, in addition to the chalice glia (as predicted by the scRNA-seq data). Tret1-1 was shown to be expressed in perineurial glia [106]. (C) Adult optic lobes expressing myrGFP driven by ltl(larval translucida)-Gal4, gene trap Trojan line, showing ltl expression in general surface glia (arrow), distal satellite (bar), and lamina surface (asterisk; fenestrated glia as predicted by the scRNA-seq data). (D, E) Maximum projection of ltl-Gal4-labelled MCFO clones showing subperineurial morphology (see Fig 2). (F) Perineurial(R85G01)>GFP adult optic lobe where CG6126 expression (magenta) was detected by in situ HCR. Most GFP positive (green) nuclei of the general perineurial glia were positive, in addition to the chalice and fenestrated glia, as predicted by the scRNA-seq data. (G) Ensheathing(R56F03)>GFP adult optic lobe where axo (axotactin) expression (magenta) was detected by in situ HCR in most GFP positive (green) nuclei adjacent to the medulla, lobula, and lobula plate neuropils. Inset of zoomed-in nuclei (scale bar is 5 μm). (H) Ensheathing(R56F03)>GFP adult optic lobe where List expression (magenta) was detected by in situ HCR in most GFP positive (green) nuclei adjacent to the medulla, lobula, and lobula plate neuropils, and in some GFP negative nuclei in the cortex area of the same neuropils (asterisk) (general cortex glia as predicted by the scRNA-seq data). (I) Ensheathing(R56F03)>GFP adult optic lobe where ana (anachronism) expression (magenta) was detected by in situ HCR in most GFP positive (green) nuclei adjacent to the neuropil as well as many nuclei in the cortex area, which were probably cortex glia based on the large size of the nuclei (predicted by scRNA- [file pbio.3002328.s016.tif]

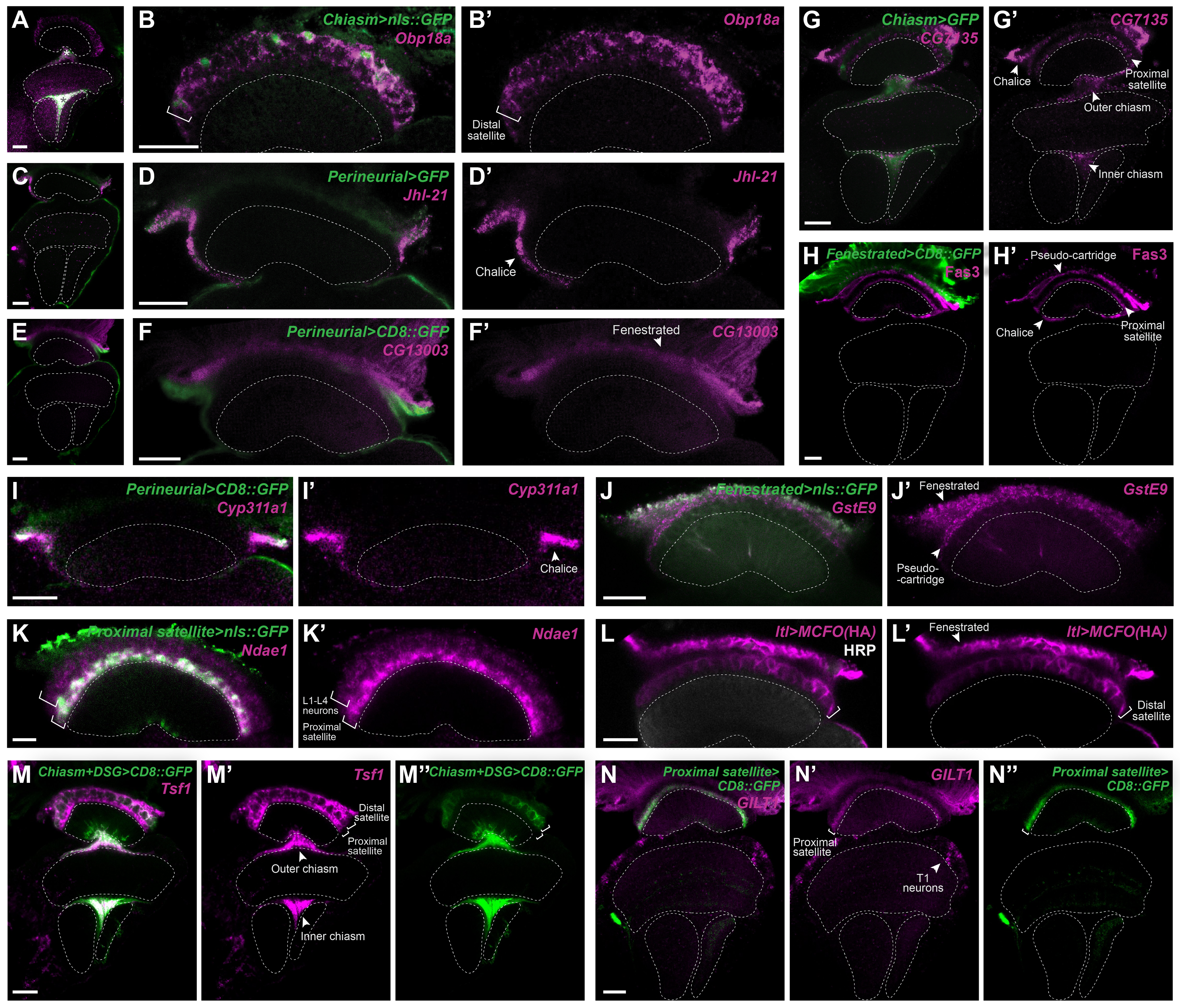

Supplement: S17 Fig — (A, B) Chiasm(R53H12)>GFP adult optic lobe, showing expression in chiasm and distal satellite glia. Obp18a expression (magenta) was detected by in situ HCR in most GFP positive (green) nuclei of chiasm and distal satellite glia. (C, D) Perineurial(R85G01)>GFP adult optic lobe where JhI-21 (Juvenile hormone Inducible-21) expression (magenta) was detected by in situ HCR, specifically in chalice glia. (E, F) Perineurial(R85G01)>CD8::GFP adult optic lobe where CG13003 expression (magenta) was detected by in situ HCR in fenestrated glia. (G) Chiasm(R53H12)>GFP adult optic lobe where CG7135 expression (magenta) was detected by in situ HCR in chiasm, chalice, and proximal satellite glia. (H) Fas3 (magenta) antibody staining of Fenestrated(R47G01)>CD8::GFP adult optic lobe, showed expression in the lamina surface glia layer below the fenestrated glia, pseudo-cartridge glia, in the chalice, and proximal satellite glia. (I) Perineurial(R85G01)>CD8::GFP adult optic lobe where Cyp311a1 (Cytochrome P450 311a1) expression (magenta) was detected by in situ HCR, specifically in chalice glia. (J) Fenestrated(R47G01)>nls::GFP adult optic lobe where GstE9 (Glutathione S transferase E9) expression (magenta) was detected by in situ HCR in fenestrated and pseudo-cartridge glia. (K) Proximal satellite(R46H12)>nls::GFP adult optic lobe where Ndae1 (Na+-driven anion exchanger 1) expression (magenta) was detected by in situ HCR in proximal satellite glia, as well as in L1, L2, L3, and L4 lamina neurons, as predicted by the neuronal scRNA-seq data from [57]. (L) Large MCFO clones labelled by ltl-Gal4, marked distal satellite (bar) and fenestrated glia (arrow). HRP in white. (M) Chiasm(R53H12)>CD8::GFP adult optic lobe where Tsf1 expression (magenta) was detected by in situ HCR in proximal and distal satellite and chiasm glia. (N) Proximal satellite(R46H12)>CD8::GFP adult optic lobe where GILT1 expression (magenta) was detected by in situ HCR in proximal satellite glia, as well as in T1 medull [file pbio.3002328.s017.tif]

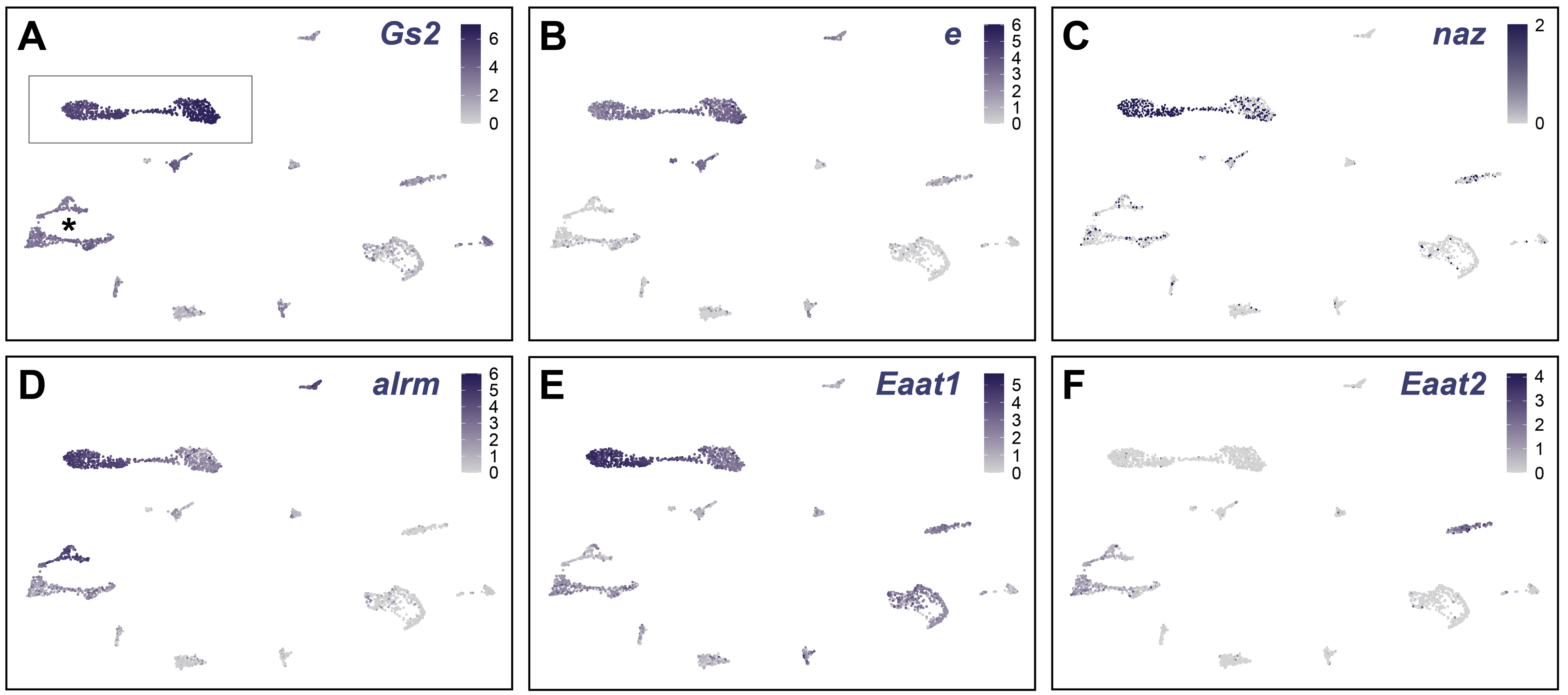

Supplement: S18 Fig — (A–C) Plots showing Gs2, e, and naz expression levels in young adult optic lobe glial clusters. Each dot represents a single cell, and the colour represents the level of expression as indicated. As previously described [107], Gs2 was expressed in both astrocytes (clusters indicated in a box in A) and ensheathing (clusters indicated with asterisk in A), while e and naz are expressed exclusively in astrocytes. (D) alrm, a known astrocyte marker [24], showed expression in the astrocyte clusters (box in A). (E) Eaat1, a known astrocyte marker [26], showed expression in the astrocyte clusters (box in A). (F) Eaat2, a known ensheathing marker [26], showed expression in the ensheathing clusters (asterisk in A) and no expression in the astrocyte clusters (box in A). The data underlying this figure can be found in S7 Data. (TIF) [file pbio.3002328.s018.tif]

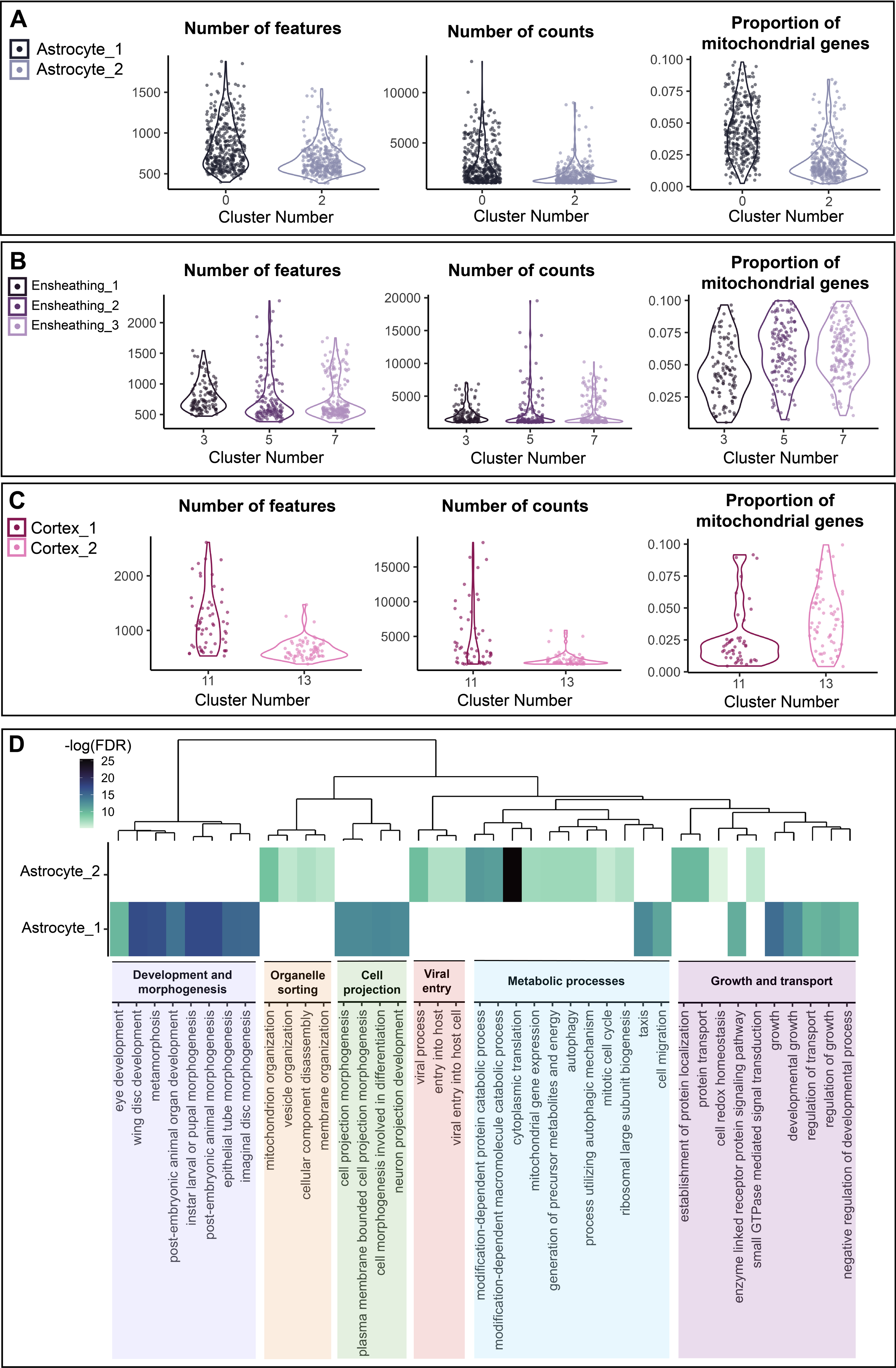

Supplement: S19 Fig — (A–C) Violin plots outlining the number of features, number of counts, and proportion of mitochondrial genes, for the astrocyte (A), ensheathing (B), and cortex (C) glia cluster multiplets. Cluster identity is colour-coded and the cluster number is indicated on the x-axes. (A) Astrocyte multiplets showed no overall trend in transcriptome quality. (B) Ensheathing glia showed a decrease in transcriptome quality in clusters #5 and #7, compared to #3. (C) Cortex glia cluster #13 appears to be of a lower quality than #11. (D) GO analysis indicating the enriched biological processes in Astrocyte_1 (cluster #0) and Astrocyte_2 (cluster #2). The data underlying this figure can be found in S7 Data and on GitHub at https://github.com/VilFernandesLab/2022_DrosophilaGlialAtlas. (TIF) [file pbio.3002328.s019.tif]
