# Supplementary material for: A Drosophila glial cell atlas reveals a mismatch between transcriptional and morphological diversity
Source: PLoS Biol. 2023 Oct 20;21(10):e3002328. doi: 10.1371/journal.pbio.3002328 (PMC10619882; doi:10.1371/journal.pbio.3002328)
Supplement: S1 File — (PDF) [file pbio.3002328.s031.pdf]

Supplementary File 1

	Surface				Neuronal cell bodies	Neuropil-associated				
	Outer		Inner			Neuropil periphery & axon tracts		Neuropil interior		
	Main	Dorsoventral channels	Main	Dorsoventral channels		Neuropil	Axon tracts	Dorsal	Lateral	Ventral
**Perineurial glia**	Surface-only perineurial glia									
	Channel-associated perineurial glia									
**Subperineurial glia**			Surface-only subperineurial glia							
			surface- and channel- associated subperineurial glia							
				channel-only subperineurial glia						
**Cortex glia**					Cortex glia					
**Ensheathing glia**						Neuropil ensheathing glia				
						Tract ensheathing glia (ensheathing/wrapping glia)				
**Astrocytes**									Type 1 astrocytes (more ramified)	
								Type 2 astrocytes (less ramified)		Type 2 astrocytes (less ramified)
