# Supplementary material for: A Drosophila glial cell atlas reveals a mismatch between transcriptional and morphological diversity
Source: PLoS Biol. 2023 Oct 20;21(10):e3002328. doi: 10.1371/journal.pbio.3002328 (PMC10619882; doi:10.1371/journal.pbio.3002328)
Supplement: S2 File — (PDF) [file pbio.3002328.s032.pdf]

Supplementary File 2

	Surface						Neuronal cell bodies			Axon tracts		Neuropil																									
	Outer surface			Inner surface								Fine processes					Highly Ramified processes																				
	Lamina		Medulla, Lobula & Lobula plate	Lamina		Medulla, Lobula & Lobula plate	Lamina		Medulla, Lobula & Lobula plate	Between lamina and Medulla		Lamina	Medulla		Lobula	Lobula plate	Lamina	Medulla										Lobula						Lobula plate			
	Distal surface	Lateral surface		Distal surface	Lateral surface		Distal (L1-L4 cell bodies)	Proximal (L5 Cell bodies)			Between Medulla, lobula and lobula plate		Distal	Lateral				1	2	3	4	5	6	7	8	9	10	6	5	4	3	2	1	1	2	3	4
Perineurial glia	Fenestrated glia																																				
		Chalice glia																																			
			General perineurial glia																																		
Subperineurial glia				Pseudo cartidge glia																																	
					Carpet glia																																
						General subperineurial glia																															
Cortex glia							Distal satellite glia																														
								Proximal satellite glia																													
									General cortex glia																												
Tract-ensheathing glia										Outer chiasm glia																											
											Inner chiasm glia																										
Ensheathing glia												Marginal glia																									
													Distal medulla ensheathing glia																								
														Lateral medulla ensheathing glia																							
															Lobula ensheathing glia																						
																Lobula plate ensheathing glia																					
Astrocytes																	Epithelial glia																				
																		Long distal medulla astrocytes																			
																		Short distal medulla astrocytes																			
																								Lateral medulla astrocyt													
																										Proximal medulla											
																												Lobula astrocytes									
																															Lobula-lobula plate astrocytes						
																																			Lobula plate astrocytes		
