# Supplementary material for: A Drosophila glial cell atlas reveals a mismatch between transcriptional and morphological diversity
Source: PLoS Biol. 2023 Oct 20;21(10):e3002328. doi: 10.1371/journal.pbio.3002328 (PMC10619882; doi:10.1371/journal.pbio.3002328)
Supplement: Attachment — Submitted filename: All reviews and responses.pdf [file pbio.3002328.s041.pdf]

Decision and reviews for your submission to eLife: 01-08-2022-TR-eLife-82347

editorial@elifesciences.org <editorial@elifesciences.org>

Mon 03/10/2022 10:04

To:Fernandes, Vilaiwan <vilaiwan.fernandes@ucl.ac.uk>

⚠ Caution: External sender

Dear Dr. Fernandes,

Thank you for submitting your article "A *Drosophila* glial cell atlas reveals that transcriptionally defined cell types can be morphologically diverse" for consideration by eLife. Your article has been reviewed by 3 peer reviewers, and the evaluation has been overseen by a Reviewing Editor and K VijayRaghavan as the Senior Editor. The following individual involved in the review of your submission has agreed to reveal their identity: Cody J Smith (Reviewer #2).

The reviewers have discussed their reviews with one another, and the Reviewing Editor has drafted this to help you prepare a revised submission.

The reviewers in the main thought that your study had great value, and that in fact should be presented as a Resource paper. Please see below and the attached reviews.

Essential revisions:
1) Reviewer 1 asks for several amendments to terminology and classification: better definition of the new perineurial glial cell type, using co-staining with a marker specific for subperineural glial cells, validate their position; address nomenclature from published studies, as referred to in the review.

2) As in Corrales et al., 2022, published nearly simultaneously with your submission, provide a list of the top number (30 is suggested) of the highest differentially expressed genes for the glial classes defined.

3) Add a number of missing citations of previous work on the topic.

4) Reviewer 2 - The Introduction should be edited and pared down.

5) A figure showing the tiling of MAP glia and apical perineurial glia would be helpful.

6) Reviewer 3 - Quantification of cell shape: even if done for some of the major categories, assess cell size, complexity, branching, volume, surface areas, etc. of the cardinal cell types, to help others know how to categorize the cells. A quantification approach would make the study even more of a resource.

7) The reviewers thought that the lack of correlations between morphotypes and transcriptional signatures might be due to sequencing depth. This should be acknowledged, your conclusion tempered, and the title modified to reflect this.

8) To this end, add a section on limitations of the study, that cautions against over-reaching

on conclusions of the paper, for example how sequencing depth could impact the conclusions.

----
eLife's editorial process also produces an assessment by peers designed to be posted alongside a preprint for the benefit of readers.

eLife Assessment:

This study presents an atlas of glial cell morphology in Drosophila, from distinct locations at different periods of life. The authors integrate morphological information with the transcriptomic signatures of those cells and find that morphological diversity among glial cells of a given class is not a strong predictor of transcriptional identity. The study is of great value as connecting morphology with scRNA sequencing analysis is rarely done and is a necessary step for understanding the underlying biology of these cells. While the weak morphotype-transcriptomic link in many cases may be due to low sequencing resolution, nonetheless, the data are of very high quality and the study will be a very useful resource for the glial biology field.

Reviewer #1 (Public Review):

In the current manuscript, scRNA data of the early "ventral nerve cord" and optic system of the adult brain are compared. The authors generated scRNAseq data for the embryo and integrated existing data sets from other labs and extracted repo-positive glial sets to present a description of the transcriptional landscape of glial cells. The main message of the paper is that morphological diversity among glial cells in a given class is not a strong predictor of transcriptional identity.

However, the data on embryonic "ventral nerve cord" glia are generated from whole embryos, and even provided that the ventral nerve cord harbors 75% of all glia and thus the majority is ventral nerve cord, the data should not be called vnc-specific. The vnc-specific data set (adult CNS) that is already published (Allen et al., 2020) is strangely not even mentioned in the current manuscript. The idea of having a comprehensive description of glial transcriptional profiles is great - but I was missing the integration of the midline glial cells, which can be considered as ensheathing glial cells that - as the cortex glia - also express wrapper (Stork et al., 2009).

Unfortunately, I found most of what is reported in this work not to be entirely new. The classification of glial diversity in the adult brain was presented by the Meinerzhagen and Gaul labs (Edwards and Meinertzhagen, 2010; Edwards et al., 2012; Kremer et al., 2017). The description of two astrocyte-like cell types is a reduction of data that defined three morphologically distinct astrocyte-like cells (Peco et al., 2016), which is not discussed. Some other aspects were ignored, too. Two other morphological distinct types of ensheathing glia exist, ensheathing glia and ensheathing/wrapping or track-associated glia were described but this is not discussed (Kremer et al., 2017; Peco et al., 2016).

Reviewer #1 (Recommendations for the authors):

The finding of a new perineurial glial cell type is interesting, but the images shown do not

document that this is indeed a perineurial glia. To show this, co-staining with a marker specific for subperineurial glial cells must be done. The newly identified perineurial cell should be sitting on the outside. Moreover, I hope that the authors do not use the name they suggested but stick to the name "channel glia", which is used for many years (Beckervordersandforth et al., 2008). Likewise, the term "apical perineurial glia" is an odd name for a cell type that is generally known as perineurial glia. If there would be a "basal perineurial glia type", yes one could use the name - but in this case please do not use new names that are not justified.

The notion that "The lamina and its associated glia lie in close proximity to an environmental interface, positioned immediately below photoreceptors of the compound eye, and were enriched for genes with immune-related function." is a misperception. All glial cells of the blood barrier share the same proximity to an environmental interface, in fact, they define the interface. All other glial cells, as well as all neurons, are well protected and should be in the same environmental compartment. Why there is an enrichment of immune-related functions? I would think this is due to a preparation artifact, and since no data are shown that would speak against this - I suggest at least rephrasing this part.

Importantly, the notion that morphological diversity is not linked to transcriptional diversity could be due to a lack of resolution in the transcriptional profiles of the different glial cells obtained in the present study. Thus, increased sequencing depth or higher cell number might lead to different results. I was also missing a discussion of the lineage relationships of the different glial cell types, which for the larval nervous system have been established a long time ago (Beckervordersandforth et al., 2008) and are known for the lamina glia, too.

Finally, a comprehensive scRNA seq analysis has just been published in Neural development (Corrales et al., 2022). Here, the Zaltic lab analyzed 130.000 CNS cells and quite nicely presented the glial clusters including an analysis of transcriptional changes during larval development. As done in this paper, the authors should present the 30 highest differentially expressed genes for all glial classes they defined.

Reviewer #2 (Public Review):

Ines Lago-Baldaia et al. investigate the connection between transcriptional and morphological diversity of glial cells. This is an important question to answer in the glial biology field and has been amplified by recent advances in single-cell sequencing. It remains unclear if transcriptional diversity that is often reported in scRNA analysis equates to morphologically distinct glia. To explore the correlation between transcriptome and morphology, the authors utilize the strength of the Drosophila model system to demonstrate that although morphotypes of glia can be identified in the nervous system, the morphotypes do not correlate with a distinct transcriptional profile. Overall, the paper is well written and the conclusion matches the results that are presented. This work will be an important contribution to the glial biology field.

Reviewer #2 (Recommendations for the authors):

This is an important topic for the field of glial biology. There have been many debates about the heterogeneity of cells within subtypes of glia. The authors present excellent data in Drosophila to show that morphotypes of glia do not correlate with transcriptional

heterogeneity that is often reported in scRNA sequencing analysis. Overall, the data is well presented and the conclusions match the data presented in the paper. Please consider these minor comments that can be addressed with changes to the text and do not require any additional experiments in revision.

Minor Comments:
The introduction is quite long and could probably be shortened significantly. I understand the importance of neurons but some of the information in the introduction is not necessary to understand the focus of this glial paper. For example, the paragraph on neuronal types provides information that, although insightful, is not needed for the readers to understand the rest of the text which is almost entirely about glia. The authors might also consider combining "the drosophila nervous" and "in the drosophila CNS" into to single paragraph that might reduce some of the redundancy of the information.

The authors note "MAP glia and apical perineurial glia tiled..." Can you add a figure panel showing that data?

The images in the figures are excellent and I commend the authors for their microscopy.

It would be helpful if the authors could propose in the discussion a few future experiments to manipulate the animal and test their hypothesis that morphotypes do not correlate with transcriptional identity. For example, if you altered the morphology of an astrocyte so that it did not interact with its typical environment, would you expect it to be transcriptionally distinct from a sister astrocyte that is interacting with its normal environment? I emphasize that the authors do not need to include these actual experiments in a revision.

There are several references where the authors use the term "first". I will leave it to the authors' discretion to keep that claim in there. However, I do not think claiming first adds much to the impact of the manuscript. The figures and results are impactful enough and do not need the "first" clarification.

Reviewer #3 (Public Review):

Our brain is comprised of both electrically active neurons that transmit information and an equal number of a set of cells called glial cells, which are actually comprised of many different cell types with a variety of functions. Compared to neurons, we know much less about glia and therefore need model systems in which they can be studied.

This study reports the generation of an atlas of glial cells in the Drosophila fly model. Drosophila glia have many similarities with those of vertebrates and are a useful model system for the interrogation of glia due to their simplicity and ease of genetic manipulation to better understand glial cell biology. This study catalogued the morphology of various types of glia in different areas of both the developing and adult fly, building a repository that will be of immense value to researchers. The study also aimed to determine how the shape of different glia, or even within glia of the same type related to the genes that they expressed, and their molecular state. The study found that while cell morphology was tightly linked to gene expression state in some cases, in others it was not, meaning that cells with very different shapes had very similar gene expression profiles/ molecular states. This latter finding suggests that at least some glial cells' shapes are more likely controlled by their interactions with the environment or molecular events that are independent of gene

expression per se. The study is very impressive in its depth of characterisation and will come to represent a very useful resource for the community of biologists who employ Drosophila to understand glial cell biology.

Reviewer #3 (Recommendations for the authors):

This study is impressive in its depth, and will no doubt be of clear benefit to the Drosophila glial cell community. I am not an expert on the analysis of RNA sequence data and so can't comment on the various technical/ protocol steps that were exhaustively explained throughout the manuscript.

To me, the manuscript is very much a resource, and that is not to diminish its importance at all. I think the annotation of cell morphologies and categorisation is important and it is wonderful that the data will be made available to the community. I do think that the cellular analyses could have been extended further. Given that the dataset is presented as an atlas, and I agree with this, it would have been very good to see some quantification of cell morphologies throughout. It was surprising to see effectively none. One could easily assess, the cell size, complexity, branching, volume, surface areas, etc. of the cardinal cell types, to help others know how to categorise their cells. IT would be interesting to know how differing glia varied in such parameters and between young and old flies (noting that the stages also represent differing areas). This is my main suggestion. For something of such great value, much more could be extracted without an undue amount of effort, and essentially no new data collection.

As I said, it will be for others to critique the clustering of molecular states/ types, but in the end, the conclusion is that molecular state does relate to cell morphology for some cells, but perhaps less so for others. One thing that has to be borne in mind is the depth of sequencing with the methods used, and that only the most highly expressed factors will be prominent, and it is of course entirely possible that lower level expression of less highly expressed factors could influence cell morphology (and indeed much more). Personally, I would de-emphasise the main pitch of the title and introduction on how morphology doesn't necessarily depend on transcriptomic profile/ state, because, a, that profile is not complete, and b, perhaps it is not that surprising for cells that are very responsive to their environment. Obviously, the same points can be made, but perhaps it would be more reflective of the core strengths of the manuscript if it were presented as a resource and one that is needed for the glial community. To me, this would still represent an important advance and it may even give the study a clearer identity rather than hanging it on the result that doesn't quite represent the entire analysis.

POSTING PUBLIC REVIEWS: Authors maintain significant control over when public reviews are posted. If you do not wish to submit a revised version of your work, please let us know, as we would be happy to delay the posting of the public reviews until after your paper has been accepted for publication in another journal.

* If you do plan to resubmit to eLife, please confirm as soon as possible by return email to say that you are happy to proceed with us posting the Evaluation Summary and Public Reviews to accompany your preprint before you resubmit (and whether you would like to

provide author responses to be posted at the same time). *

If you believe there are clear factual errors in these sections that preclude public posting, please let us know. Any sections marked as "Essential Revisions" or "Recommendations for the authors" will NOT be posted to the preprint.

Information about our revision policy: As the editors have judged that your manuscript is of interest, but additional experiments are required before it is published, we are giving authors as much time as they need to submit revised manuscripts.

Information to help as you revise your submission:

* We will look forward to hearing from you with a revised article with tracked changes, and a response letter (uploaded as an editable file) describing the changes made in response to the decision and review comments.

* If source manuscript files have not already been provided (including figures in an RGB color space, uploaded individually), we will need them at the revision stage; further details are here: https://submit.elifesciences.org/html/elife_author_instructions.html#revised

* If your study includes bench research, please include a Key Resources Table within your resubmission. This is designed to highlight genetically modified organisms and strains, cell lines, reagents, and software that are essential to reproduce the results presented (but it is not designed to be a comprehensive list of all the materials and resources used).You should download the following template, which includes notes on completion as well as an example table: [https://elife-cdn.s3.amazonaws.com/author-guide/elifeKRTtemplatev2.docx] For relevant submissions, the completed table should be incorporated within your article file at the very beginning of the Materials and Methods section (examples in published articles are available at [https://elifesciences.org/articles/44199#s4] and [https://elifesciences.org/articles/45342#s4]).

* If your work involved the use of cell lines, please indicate in the Materials and Methods section of your manuscript if their identity has been authenticated, state the authentication method (such as STR profiling), and report the mycoplasma contamination testing status. Please consult our Journal Policies for further details: https://submit.elifesciences.org/html/elife_author_instructions.html#policies

* Advice about statistical reporting: in your revised paper, please ensure that any statistical analysis methods have been described and justified; ensure that raw data are presented in figures whenever informative to do so (typically when N per group is less than 10); for each experiment, ensure that you have identified the statistical tests used, the exact values of N, definitions of center, methods of multiple test correction, and dispersion and precision measures; and please report exact p-values wherever possible alongside the summary statistics and 95% confidence intervals (for all key questions and not only when the p-value is less than 0.05).

* If your revised article is accepted for publication, please note that authors can choose to

have their accepted PDF published by eLife within a few days of acceptance, in advance of the version of record that will be subject to technical processing, editing, and author proofing. Please consider whether this is suitable for your work, so you can select the appropriate option at the time of resubmission. In the event that the study has potentially broad public relevance, we recommend you consult your press officer before making this decision.

Please use the following link when you are ready to resubmit:

[https://submit.elifesciences.org/cgi-bin/main.plex?el=A4Hz7oth6A3BRJo1I6A9ftdPfFecXQK1XKlU7W1wToNgZ]

Best wishes,

Carol Mason
Reviewing Editor, eLife
Vijay
K VijayRaghavan
Senior Editor, eLife

-----
eLife Sciences Publications, Ltd is a limited liability non-profit non-stock corporation incorporated in the State of Delaware, USA, with company number 5030732, and is registered in the UK with company number FC030576 and branch number BR015634 at the address Westbrook Centre, Milton Road, Cambridge, CB4 1YG, UK.

We are grateful to the reviewers for their constructive feedback and suggestions. Overall, the reviewers felt that the work was impressive in its depth and of great value as a community resource. We have incorporated reviewer feedback and addressed concerns, which has improved the manuscript greatly.

Essential revisions:

1. Reviewer 1 asks for several amendments to terminology and classification: better definition of the new perineurial glial cell type, using co-staining with a marker specific for subperineural glial cells, validate their position; address nomenclature from published studies, as referred to in the review.
   - Reviewer 1 was concerned that the glial subclass we referred to as midline-associated perineurial glia (MAP glia) were in fact the 'channel glia', previously described as a subperineurial glial subclass (Beckervordersandforth et al., 2008; Ito et al., 1995). We are grateful to Reviewer 1 for drawing our attention to the channel (subperineurial) glia, which we had missed previously.
   - To be consistent with prior terminology we now refer to the MAP glia as 'channel perineurial glia' and clarify the previously named channel glia as 'channel subperineurial glia'.
   - We present several lines of evidence to support the notion that the previously named MAP glia are a perineurial and not a subperineurial glial subclass:
      i. We identified the 'channel subperineurial glia', which we had missed previously and show that these display a distinct morphology to 'channel peineurial glia' (previously MAP glia) (Figure 1B-F). Both subclasses of subperineurial glia were previously reported to express *moody* (Beckervordersandforth et al., 2008). When we generated MCFO clones with higher frequency using *moody-Gal4* we recovered clones with morphologies belonging to both subperineurial glial subclasses, *i.e.,* surface-only subperineurial glia and channel subperineurial glia (Figure 1G-L). Importantly, channel subperineurial glia still differed in morphology from the channel perineurial glia (previously MAP glia) (Figure 1B-F compared to Figure 1G-L).
      ii. We were unable to obtain an aliquot of the Moody antibody, which is not commercially available, to check that channel perineurial glia (previously MAP glia) do not express this subperineurial glia marker. However, we did not recover *moody-Gal4* MCFO clones in these cells and when we viewed individual channel perineurial glial cells in a genetic background where all glial cells were labelled (*repo-lexA>lexAop-myr::tdTomato*) we observed that they clearly occupied the outer glial surface (Figure 1 – figure supplement 1B,C).
      iii. Finally, we show that the channel perineurial glia, which also associate with the dorsoventral channels, tile with surface-only perineurial glia (Figure 1B,C,E,F) and that the surface-only subperineurial glia tile with the channel subperineurial glia (Figure 1H,I and Figure 4E).

- Together these data argue that the glia we previously referred to as MAP glia are indeed a subclass of perineurial glia. For consistency with previous terminology we now refer to these as "channel perineurial glia". Thus like the main surface of the VNC, the dorsoventral channels are also associated with perineurial and subperineurial glia.

2. As in Corrales et al., 2022, published nearly simultaneously with your submission, provide a list of the top number (30 is suggested) of the highest differentially expressed genes for the glial classes defined.
   - We now include lists of genes with enriched expression in each transcriptionally defined glial type (including midline glia) as:
     - Source data file 1 (for midline glia)
     - Source data file 2 (for embryonic *repo*+ glia)
     - Source data file 3 (for adult optic lobe glia)

3. Add a number of missing citations of previous work on the topic.
   - We apologise for these omissions and have added in citations raised by Reviewer 1. Namely, Ito et al., 1995, Beckervordersandforth et al., 2008, Allen et al., 2020 and Corrales et al., 2022.

4. Reviewer 2 - The Introduction should be edited and pared down.
   - We have removed the section on neuronal diversity and redundant sections on Drosophila glia. We trimmed corresponding sections in the discussion.

5. A figure showing the tiling of MAP glia and apical perineurial glia would be helpful.
   - This is now included, please see response to (1).

6. Reviewer 3 - Quantification of cell shape: even if done for some of the major categories, assess cell size, complexity, branching, volume, surface areas, etc. of the cardinal cell types, to help others know how to categorize the cells. A quantification approach would make the study even more of a resource.
   - We focused on quantifying astrocyte morphologies as astrocytes represent the glial class which harbours the most morphological diversity and because of the interest in astrocyte morphological diversity in vertebrates. We have included these characterizations in Figure 1 – figure supplement 2 and Figure 2 – figure supplement 2 for the embryo and adult optic lobe, respectively. We also provide 3D reconstructions of the different astrocyte morphological categories in Videos 1-11.
   - In addition, we include two tables as supplementary files to serve as a classification key for distinguishing between the different glial classes and morphological categories based on position and neuronal association.
     - Supplementary file 1 for embryonic Repo+ glia key
     - Supplementary file 2 for Adult Optic lobe glia key

- These data have improved our manuscript and made it more valuable as a community resource. We are grateful for this suggestion.

7. The reviewers thought that the lack of correlations between morphotypes and transcriptional signatures might be due to sequencing depth. This should be acknowledged, your conclusion tempered, and the title modified to reflect this. To this end, add a section on limitations of the study, that cautions against over-reaching on conclusions of the paper, for example how sequencing depth could impact the conclusions.
   - We have now added a section on the limitations of the study as part of the discussion, in which we highlight that caution must be employed in interpreting our results given the depth of sequencing constraints of current single cell RNA sequencing approaches. We also temper our conclusions to refer to "detectable transcriptional diversity" throughout the manuscript, again emphasizing that we may be limited by what we are able to detect.
   - As well, we have modified the title to: "A Drosophila glial cell atlas reveals a mismatch between detectable transcriptional diversity and morphological diversity"

Reviewer #1:

8. The data on embryonic "ventral nerve cord" glia are generated from whole embryos, and even provided that the ventral nerve cord harbors 75% of all glia and thus the majority is ventral nerve cord, the data should not be called vnc-specific. The vnc-specific data set (adult CNS) that is already published (Allen et al., 2020) is strangely not even mentioned in the current manuscript. The idea of having a comprehensive description of glial transcriptional profiles is great - but I was missing the integration of the midline glial cells, which can be considered as ensheathing glial cells that - as the cortex glia - also express wrapper (Stork et al., 2009).
   - We agree with Reviewer 1 that the embryonic glia dataset represents all glia and not just VNC glia. We have amended the text accordingly.
   - We now cite the Allen et al., 2020. Apologies for this omission.
   - **Midline Glia:**
     - The embryonic glial cells analysed in the previous version of our manuscript included only *repo*+ glia only and therefore did not include midline glia, which do not express *repo* (Jacobs, 2000). In the revised manuscript, we reanalysed the complete embryonic dataset and identified the midline glia based on known markers and *in vivo* validation (Figure 3 – figure supplement 1). We also provide a list of genes that show enriched expression in the midline glial cluster as a supplementary file (Source data file 1).
     - We performed hierarchical cluster analysis on midline glia, all embryonic *repo*+ glial clusters and embryonic neuronal clusters to

determine the relationship of midline glia to other glia. Interestingly, midline glia formed an outgroup to both neurons and *repo*+ glia (Figure 3 – figure supplement 1F), suggesting that they are quite distinct from other (*repo*+) glial classes. This is expected given their mesectodermal origin (Kosman et al., 1991; Thomas et al., 1988). Indeed, although midline glia express wrapper, otherwise known as a cortex glia marker (Banerjee et al., 2017; Noordermeer et al., 1998; Stork et al., 2009), they do not resemble cortex glia in form or function but instead ensheath commissural axons and play critical roles in axon guidance and VNC morphogenesis (Jacobs, 2000). Midline glia have been characterised extensively by several groups (Hartenstein, 2011; Hidalgo, 2003; Jacobs, 2000; Kearney et al., 2004; Vasenkova et al., 2006; Wheeler et al., 2006), therefore, given their distinct origin and the ambiguity surrounding their functional classification, we instead focused our analyses on *repo*+ glia in this manuscript.

9. Unfortunately, I found most of what is reported in this work not to be entirely new. The classification of glial diversity in the adult brain was presented by the Meinerzhagen and Gaul labs (Edwards and Meinertzhagen, 2010; Edwards et al., 2012; Kremer et al., 2017). The description of two astrocyte-like cell types is a reduction of data that defined three morphologically distinct astrocyte-like cells (Peco et al., 2016), which is not discussed. Some other aspects were ignored, too. Two other morphological distinct types of ensheathing glia exist, ensheathing glia and ensheathing/wrapping or track-associated glia were described but this is not discussed (Kremer et al., 2017; Peco et al., 2016).
   - We respectfully disagree with Reviewer 1's assessment that much of the work presented in not new. This work represents the first *Drosophila* glial cell atlas with thorough validation of cluster marker expression *in vivo*. It is also the first <u>systematic exploration of the relationship between glial morphology and transcriptional signature</u>, a controversial topic in the field of glial biology. We fully agree that much of the adult glial morphology had been characterised previously by the Meinerzhagen and Gaul labs among many others and we acknowledge this explicitly in our manuscript and in references to Figures 2 (one out of a total of 9 main figures). Indeed, it is because *Drosophila* glial morphology has been so well characterised that a comprehensive exploration of the relationship between morphology and transcriptional signature was even feasible. Moreover, our revised manuscript also provides more in-depth morphological characterisation and quantification of glial morphology and defines subclasses and morphologies not described previously (*e.g.* channel perineurial glia and astrocyte morphologies of the lobula and lobula plate). Indeed, even the channel subperineurial glia, which were identified based on lineage relationships, nuclear position and molecular markers, were not described in morphological terms.

- The 3 distinct astrocyte populations defined in Peco et al., (2016) refer to cell body position and neuropil domains covered by astrocytes. We now include this categorisation in our quantification of astrocyte morphology (See response to (6) and Figure 1 – figure supplement 2) and discuss their relationship to the type 1 and type 2 astrocyte morphologies that we observed.
- As well we now include the ensheathing/wrapping or tract ensheathing glia as a morphological category of ensheathing glia in the manuscript (Figure 1A,N,O).

10. The finding of a new perineurial glial cell type is interesting, but the images shown do not document that this is indeed a perineurial glia. To show this, co-staining with a marker specific for subperineurial glial cells must be done. The newly identified perineurial cell should be sitting on the outside. Moreover, I hope that the authors do not use the name they suggested but stick to the name "channel glia", which is used for many years (Beckervordersandforth et al., 2008). Likewise, the term "apical perineurial glia" is an odd name for a cell type that is generally known as perineurial glia. If there would be a "basal perineurial glia type", yes one could use the name - but in this case please do not use new names that are not justified.
    - Please see response to (1).

11. The notion that "The lamina and its associated glia lie in close proximity to an environmental interface, positioned immediately below photoreceptors of the compound eye, and were enriched for genes with immune-related function." is a misperception. All glial cells of the blood barrier share the same proximity to an environmental interface, in fact, they define the interface. All other glial cells, as well as all neurons, are well protected and should be in the same environmental compartment. Why there is an enrichment of immune-related functions? I would think this is due to a preparation artifact, and since no data are shown that would speak against this - I suggest at least rephrasing this part.
    - The entire surface of the CNS is indeed covered by surface or 'blood brain barrier' (BBB) glia. However, the surface glia that cover the lamina are densely perforated to allow the photoreceptor axons to enter the optic lobe. This is even reflected in nomenclature (*e.g.* 'fenestrated' glia). Thus, the BBB over the lamina is quite distinct from the BBB over any other region of the CNS.
    - We think it unlikely that the enrichment for immune-related gene expression that we observe in lamina glia relative to general glia is due to a preparation artefact (*i.e.*, artifact of dissociation) since dissociation artefacts are generally associated with the cellular stress response rather than the immune response. We did not observe an enrichment of stress-related gene expression in lamina glia.

- Furthermore, among the markers we validated *in vivo* as part of our efforts to annotate transcriptional clusters were *Tsf1, GILT1* and *JhI-21*, which were enriched in lamina glial clusters and which are known to be involved in the antibacterial and/or antifungal immune response (Figure 5 – figure supplement 4D, M,N)(Jin et al., 2008; Kongton et al., 2014; Latsenko et al., 2020; Weber et al., 2022). These validations were performed in intact optic lobes that were fixed no more than 15 minutes after dissection, thus making it unlikely that the expression of these genes is due to a preparation artefact. We highlight these data better in the revised manuscript.

12. Importantly, the notion that morphological diversity is not linked to transcriptional diversity could be due to a lack of resolution in the transcriptional profiles of the different glial cells obtained in the present study. Thus, increased sequencing depth or higher cell number might lead to different results.
    - Please see response to (7) above.

13. I was also missing a discussion of the lineage relationships of the different glial cell types, which for the larval nervous system have been established a long time ago (Beckervordersandforth et al., 2008) and are known for the lamina glia, too.
    - We agree that exploring how or if lineage relationships are reflected in similarities in expression profiles and/or morphology would be very interesting, however, in this study, we chose to focus just on the relationship between morphology and transcriptional profile, as the first aim of the manuscript was simply to annotate fly glia and provide an atlas. Thus, exploring lineage relationships is beyond the scope of this study. Nonetheless, we cite Beckervordersandforth et al., 2008 as well as Edwards et al., 2012 throughout our manuscript and suggest exploring lineage relationships in our discussion.

14. Finally, a comprehensive scRNA seq analysis has just been published in Neural development (Corrales et al., 2022). Here, the Zaltic lab analyzed 130.000 CNS cells and quite nicely presented the glial clusters including an analysis of transcriptional changes during larval development. As done in this paper, the authors should present the 30 highest differentially expressed genes for all glial classes they defined.
    - Please see response to (2).

Reviewer #2:

15. The introduction is quite long and could probably be shortened significantly. I understand the importance of neurons but some of the information in the

introduction is not necessary to understand the focus of this glial paper. For example, the paragraph on neuronal types provides information that, although insightful, is not needed for the readers to understand the rest of the text which is almost entirely about glia. The authors might also consider combining "the drosophila nervous" and "in the drosophila CNS" into to single paragraph that might reduce some of the redundancy of the information.

- We have removed the section on neuronal diversity and redundant sections on Drosophila glia. Doing so has improved the readability of the manuscript immensely.

16. The authors note "MAP glia and apical perineurial glia tiled..." Can you add a figure panel showing that data?
    - Please see response to (1) and (5) above.

17. It would be helpful if the authors could propose in the discussion a few future experiments to manipulate the animal and test their hypothesis that morphotypes do not correlate with transcriptional identity. For example, if you altered the morphology of an astrocyte so that it did not interact with its typical environment, would you expect it to be transcriptionally distinct from a sister astrocyte that is interacting with its normal environment? I emphasize that the authors do not need to include these actual experiments in a revision.
    - We now include a few experiments in the discussion to test how equivalent glia might alter their morphology and transcriptional profiles in distinct environments.
        - "In the future, one could leverage genetic manipulations that alter neuropil structure to assess how equivalent glia might alter their morphology and transcriptional profiles in distinct environments. For example, one could assess glial morphologies and transcriptional profiles while (i) altering neuronal diversity and composition by manipulating the temporal series in medulla neuroblasts (Erclik et al., 2017; Konstantinides et al., 2021; Li et al., 2013; Zhu et al., 2022), or by blocking lamina neuron differentiation (Fernandes et al., 2017; Prasad et al., 2021), or (ii) by modifying neuropil stratifications with genetic perturbations that disrupt neuronal targeting (Zhang et al., n.d.)."

18. There are several references where the authors use the term "first". I will leave it to the authors' discretion to keep that claim in there. However, I do not think claiming first adds much to the impact of the manuscript. The figures and results are impactful enough and do not need the "first" clarification.
    - Thank you, yes, we've amended the text accordingly.

Reviewer #3:

19. This study is impressive in its depth, and will no doubt be of clear benefit to the Drosophila glial cell community. I am not an expert on the analysis of RNA sequence data and so can't comment on the various technical/ protocol steps that were exhaustively explained throughout the manuscript.
    To me, the manuscript is very much a resource, and that is not to diminish its importance at all. I think the annotation of cell morphologies and categorisation is important and it is wonderful that the data will be made available to the community. I do think that the cellular analyses could have been extended further. Given that the dataset is presented as an atlas, and I agree with this, it would have been very good to see some quantification of cell morphologies throughout. It was surprising to see effectively none. One could easily assess, the cell size, complexity, branching, volume, surface areas, etc. of the cardinal cell types, to help others know how to categorise their cells. It would be interesting to know how differing glia varied in such parameters and between young and old flies (noting that the stages also represent differing areas). This is my main suggestion. For something of such great value, much more could be extracted without an undue amount of effort, and essentially no new data collection.
    - Thank you for this suggestion. Please see response to (6).

20. As I said, it will be for others to critique the clustering of molecular states/ types, but in the end, the conclusion is that molecular state does relate to cell morphology for some cells, but perhaps less so for others. One thing that has to be borne in mind is the depth of sequencing with the methods used, and that only the most highly expressed factors will be prominent, and it is of course entirely possible that lower level expression of less highly expressed factors could influence cell morphology (and indeed much more). Personally, I would de-emphasise the main pitch of the title and introduction on how morphology doesn't necessarily depend on transcriptomic profile/ state, because, a, that profile is not complete, and b, perhaps it is not that surprising for cells that are very responsive to their environment. Obviously, the same points can be made, but perhaps it would be more reflective of the core strengths of the manuscript if it were presented as a resource and one that is needed for the glial community. To me, this would still represent an important advance and it may even give the study a clearer identity rather than hanging it on the result that doesn't quite represent the entire analysis.
    - We agree that this work will be an important resource for the community and have submitted it as a resource.
    - Please see response to (7). We have added a section on the limitations of the study, in which we highlight all the caveats that the reviewers raised.
    - We would like to clarify that the conclusion we are drawing from our data is that glial morphological diversity does not correlate with transcriptional diversity, within the current limits of detection. Indeed, the fact that only glial classes and subclasses associated with the lamina (compared to all other

neuropils) could be transcriptionally defined, argues to us that these cells are transcriptionally distinct not because of their distinct morphologies but because of their association with the lamina. We apologise for not articulating this logic clearly in the previous manuscript.

- Given the ambiguity surrounding the meaning of ‘heterogeneity’ in the field of glial biology, we do feel that it is important to highlight this interpretation while also drawing attention to all the associated caveats and limitations of our study.

eLife decision: 01-08-2022-TR-eLife-82347R1

editorial@elifesciences.org <editorial@elifesciences.org>
Fri 16/06/2023 09:51
To:Fernandes, Vilaiwan <vilaiwan.fernandes@ucl.ac.uk>

⚠ Caution: External sender

Dear Dr Fernandes,

Thank you for resubmitting your work entitled "A *Drosophila* glial cell atlas reveals a mismatch between detectable transcriptional diversity and morphological diversity" for further consideration by eLife. Your revised article has been evaluated by K VijayRaghavan (Senior Editor) and a Reviewing Editor.

The manuscript has been improved but there are some remaining issues raised by Reviewer 1 that need to be addressed, as outlined below:

Reviewer 1 thought that the integration of previous RNAseq data sets into a common glia-focused data set will be a useful resource, but raised several criticisms and claims made, especially with reference to claims of new morphologically and transcriptionally distinct surface glial populations in the ventral nerve cord. Most can be remediated textually.

1. CG5080-Gal4 was used to generate MCFO clones, but the following statement is misleading:"Previous reports identified two classes of surface glia, perineurial on the outer surface of the VNC (CG5080+) and subperineurial glia (moody+) lying below perineurial glia (Freeman, 2015; Pogodalla et al., 2022)." In both papers cited, perineurial glia are not defined as CG5080+. Please adjust.

2. The reviewer did not find previous references that CG5080-Gal4 is perineurial glia-specific.

a. Beckervordersandforth (2008, Table 3) shows that all glial cells that are CG5080 positive are also moody positive (moody being an established SPG marker). The MCFO clones presented, indeed, support the notion that CG5080 cells are subperineurial glial cells.

b. Perineurial glial cells are well known in the field and several Gal4 drivers have been used in the past to direct expression in these glial cells. In previous studies (Awasaki et al., 2008; Stork et al., 2008) perineurial glial cells were characterized as partially overlapping cells with many fine small processes - which are not visible in the images shown in this paper
.
c. As for the comment that anti-Moody antibody is not available: there is a commercially available antibody against Mdr65 which gives an excellent and very specific staining as published by the Bainton lab. Moreover, even in the absence of an antibody, the combination of (moody-lexA, lexAop-GFP, CG5080-Gal4, UAS-tdTomato) should allow definition of whether the respective cells are identical or not.

3. The reviewer takes issue with the statement: "the gene wrapper, which is a known cell type-specific marker of cortex glia (Coutinho-Budd et al., 2017)". Instead, Coutinho-Budd

used a promoter fragment of the gene wrapper, which is specific for cortex glia. In Noordermeer et al. (1998) wrapper is described by protein expression as being midline specific in the embryo. BDGP in situ data show few lateral cells during embryogenesis but these cells are not classified as cortex glia.

4. In addition, this reviewer believes that validation of expression patterns using enhancer-Gal4 constructs is generally problematic. CRIMIC or Trojan-Gal4 insertions should be used. please respond with specific arguments about using the constructs you used versus these insertions.

5. Pippin is reported to be expressed in perineurial and subperineurial glia (McMullen et al 2021); please cite this reference.

Lastly, Reviewer 3 thought that the word "detectable" in the title was not necessary, and we agree.

We will look forward to hearing from you with a revised article and a response letter describing the changes made. We hope you will resubmit in a timely manner for further consideration and you can use the following link when you are ready to resubmit:

[https://submit.elifesciences.org/cgi-bin/main.plex?el=A5Hz7oth7B5BRJo6I5A9ftdPfFecXQK1XKlU7W1wToNgZ]

Best wishes,

Carol Mason
Reviewing Editor, eLife

K VijayRaghavan
Senior Editor, eLife

-----
eLife Sciences Publications, Ltd is a limited liability non-profit non-stock corporation incorporated in the State of Delaware, USA, with company number 5030732, and is registered in the UK with company number FC030576 and branch number BR015634 at the address Westbrook Centre, Milton Road, Cambridge, CB4 1YG, UK.

Thank you for your feedback on our revised manuscript. Below we address the points raised by the reviewers.

1. CG5080-Gal4 was used to generate MCFO clones, but the following statement is misleading:"Previous reports identified two classes of surface glia, perineurial on the outer surface of the VNC (CG5080+) and subperineurial glia (moody+) lying below perineurial glia (Freeman, 2015; Pogodalla et al., 2022)." In both papers cited, perineurial glia are not defined as CG5080+. Please adjust.

- We apologise for this citation error. *cg5080* was previously reported to be a marker of surface glia without distinguishing which class (perineurial or subperineurial) expressed it (Beckervordersandforth et al., 2008; DeSalvo et al., 2014), therefore we have modified the text as follows:
  Page 5:
  "The surface glia comprise two glial classes, perineurial on the outer surface of the VNC and subperineurial glia positioned below perineurial glia (reviewed in Freeman, 2015). Early glial studies did not distinguish between these two classes and reported expression of the markers *cg5080* and *moody* in surface-associated glia, a result also confirmed by bulk RNA profiling of surface glia (Beckervordersandforth et al., 2008; DeSalvo et al., 2014; Ito et al., 1995). Later, Moody was shown to be expressed in subperineurial glia exclusively (Bainton et al., 2005; Schwabe et al., 2005). To analyse surface glia morphology in the VNC, we used the *CG5080-Gal4* and *moody-Gal4* drivers to generate MCFO clones."

2. The reviewer did not find previous references that CG5080-Gal4 is perineurial glia-specific.

- We apologise for our citation error and have amended the text as indicated above.

a. Beckervordersandforth (2008, Table 3) shows that all glial cells that are CG5080 positive are also moody positive (moody being an established SPG marker). The MCFO clones presented, indeed, support the notion that CG5080 cells are subperineurial glial cells.

- Early studies did not distinguish between perineurial and sub-perineurial glia, instead Ito et al. (1995) state that: *"The "surface-associated glia" category represents the cells that are closely associated with the CNS surface. The surface is covered by the perineurium, which consists of the acellular neural lamella (Scharrer 1939) and the surface-associated glial cells underneath (Fig. 3). In the embryonic and L1 VNC we defined two subtypes of this glial category. The "subperineurial glia" (SPG) lie beneath the outer surface of the VNC, and the "channel glia" (CG) lie along the dorsoventral channels".* Along similar lines, in 2008 Beckervordersandforth et al. state, *"The category of surface-associated glia includes two subgroups: the subperineurial glia (SPG) that lie underneath the outer surface of the CNS, and the channel glia (CG) which are positioned along the dorsoventral channels".*
  In other words, in these early reports "subperineurial glia" (SPG) include what the field now knows to be both the perineurial and subperineurial glial classes.
- In Table 3 of Beckervordersandforth et al. (2008), the authors state that *cg5080* and *moody* are expressed in "SPG" (i.e. both classes of surface glia) in <u>50–100% of examined hemisegments</u>. Therefore, we cannot draw conclusions as to whether these expression patterns refer to perineurial glia, subperineurial glia or both, nor whether these markers were evaluated and observed in the same cells as representative images were not included in Beckervordersandforth et al. (2008).
- We used *CG5080-Gal4* and other drivers in the experiments shown in Figure 1 to characterise the morphological diversity of glia in the early L1 VNC, not to define or validate marker expression. We therefore used available Gal4 lines that were known to target different glial classes to generate MCFO clones and analysed their morphology. These experiments showed that *CG5080-Gal4* does indeed label perineurial glia, as assessed by position and morphology (more details below).

b. Perineurial glial cells are well known in the field and several Gal4 drivers have been used in the

past to direct expression in these glial cells. In previous studies (Awasaki et al., 2008; Stork et al., 2008) perineurial glial cells were characterized as partially overlapping cells with many fine small processes - which are not visible in the images shown in this paper.

- We have updated Figure 1 (see below) to include single channel insets to better highlight the fine processes, fibrous morphology and loose tiling(partial overlap) of perineurial glial clones labelled by *CG5080-Gal4*. Awasaki et al. (2008) reported that perineurial and subperineurial glia can be readily distinguished by size alone, with perineurial glia appearing smaller and more numerous than subperineurial glia. Consistent with this, we found that cells labelled by *CG5080-Gal4* MCFO were smaller and more closely packed compared to *moody-Gal4* MCFO clones (Figure 1B-L). As well, Kremer et al. (2017) described subperineurial glia as containing “many ring-like membranous structures within the cells [which] reveal themselves in cross section as cap-like basal protrusions that cover individual neuronal cell bodies within the cortex”. We observe the same in our subperineurial glial (*moody-Gal4*) clones (honeycomb-like pattern in Figure 1G inset and 1H) but not in *CG5080-Gal4* MCFO clones (Figure 1B,E).
- Finally, it is important to note that cell morphologies do not match perfectly between different developmental stages or brain regions. Even for the examples referred to by Reviewer 1, Stork et al. (2008) reported numerous fine processes for perineurial glia in L3 larvae, whereas Awasaki et al. (2008) did not report the same for perineurial glia in adults (See below).

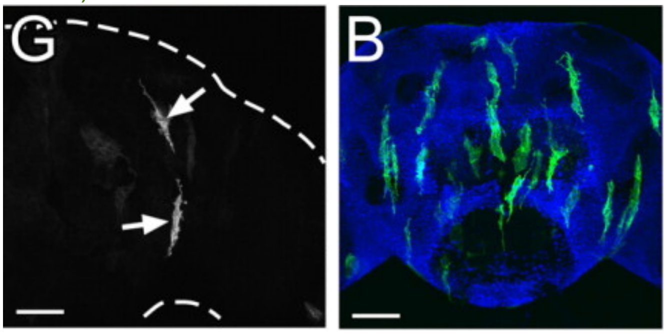

In Awasaki et al., 2008 (https://www.ncbi.nlm.nih.gov/pmc/articles/PMC6671902/): Panel G from Figure 1 and Panel B from Figure 2 show perineurial glia in the adult CNS, which are described as small, slender and oblong.

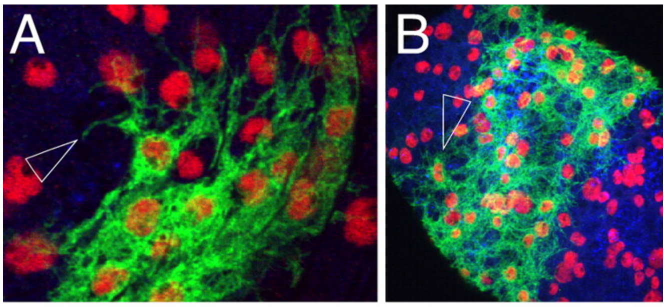

In Stork et al., 2008 (https://www.jneurosci.org/content/28/3/587.long): Panels A and B from Figure 2 show perineurial glia labelled by *repo-Gal4* and described as a “sheath of astrocyte-like shaped cells” and possessing a “star-like shape with many thin cell protrusions”.

These differences are likely attributed to differences in developmental stage and/or brain region. Thus, for our analysis we use a combination of features to define a particular glial class as detailed above and are confident in our assertion that the *CG5080-Gal4* clones presented in Figure 1B-F correspond to perineurial and not subperineurial glia.

Figure 1

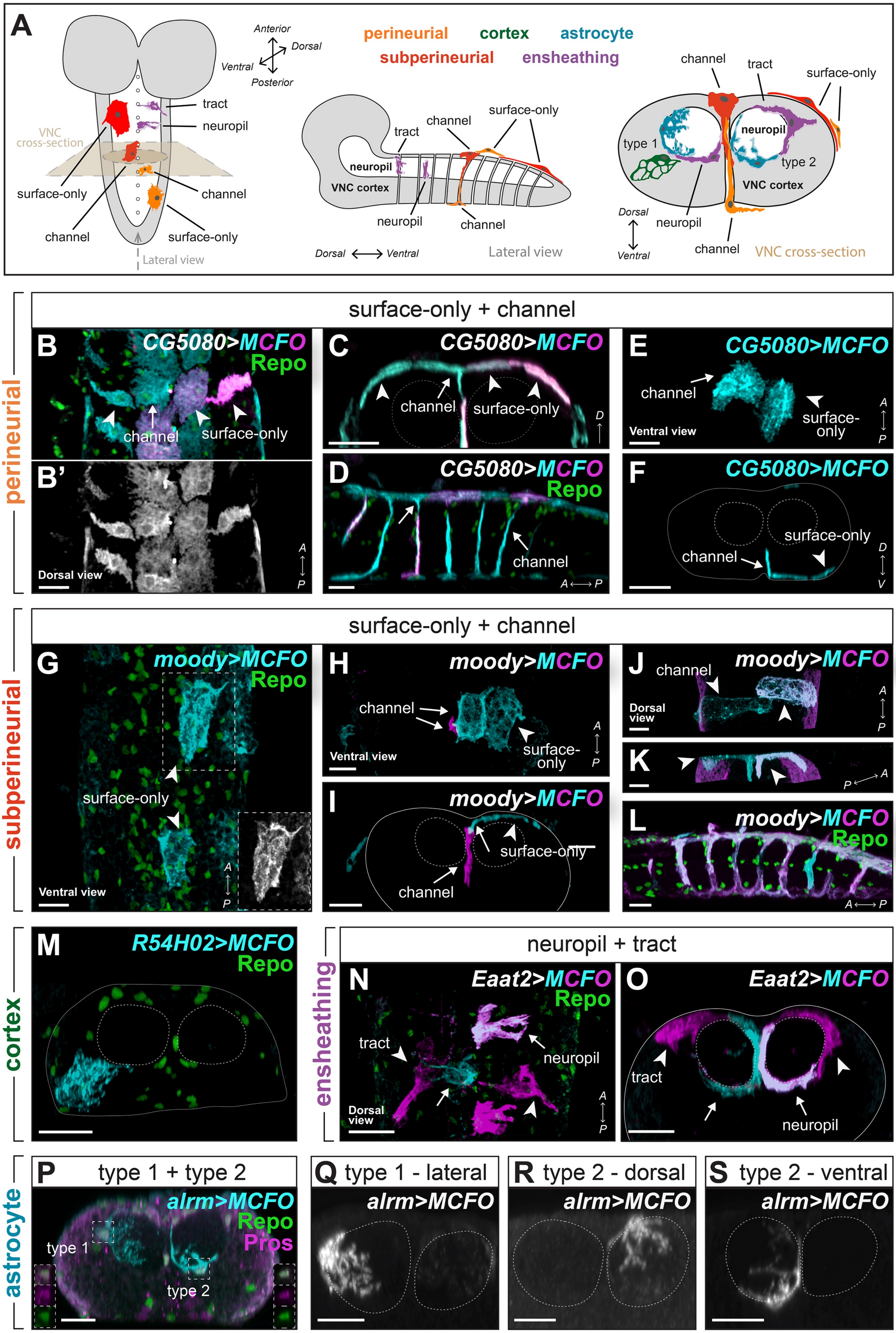

A
Anterior
Dorsal
Ventral
Posterior
perineurial
cortex
astrocyte
subperineurial
ensheathing
tract
neuropil
VNC cross-section
surface-only
channel
Lateral view
VNC cortex
Dorsal ⟷ Ventral
type 1
type 2
perineurial
surface-only + channel
B CG5080>MCFO Repo
B'
Dorsal view
C CG5080>MCFO
D CG5080>MCFO Repo
E CG5080>MCFO
Ventral view
F CG5080>MCFO
subperineurial
G moody>MCFO Repo
H moody>MCFO
I moody>MCFO
J moody>MCFO
K
L moody>MCFO Repo
cortex
M R54H02>MCFO Repo
ensheathing
neuropil + tract
N Eaat2>MCFO Repo
O Eaat2>MCFO
astrocyte
P type 1 + type 2
alrm>MCFO Repo Pros
Q type 1 - lateral
R type 2 - dorsal
S type 2 - ventral
alrm>MCFO

Figure 1. Morphologies of newly hatched VNC glia.
(A) Schematics of the embryonic CNS along different axes with the five glial classes indicated. Cortex (neuronal cell bodies) indicated in grey.
(B) Dorsal surface view of the VNC showing surface-only and channel perineurial and subperineurial glial cells on the surface with characteristic fibrous morphology and loose tiling.
(C) Cross-sectional view of the VNC showing the same cells as in (B) now showing the tapered projection from a (dorsal) channel perineurial glial cell that tiles with neighbouring surface-only perineurial glial cells.
(D) Lateral view of the VNC showing channel perineurial glia on the ventral and dorsal surfaces, each sending a single projection with ventral channel perineurial glia sending longer processes than their dorsal counterparts.
(E) Ventral surface view of the VNC showing surface-only and channel perineurial and subperineurial glial that tile with each other loosely.
(F) Cross-sectional view of the VNC showing the same cells as in (C) now showing the tapered projection from a (dorsal) channel perineurial glial cell that tiles with a neighbouring surface-only perineurial glial cell.
(G) Surface view of the VNC showing polygonal-shaped surface-only subperineurial glia on the surface.
(H) Surface view of the VNC showing polygonal-shaped surface-only subperineurial glia (cyan) on the surface and an underlying channel-only subperineurial glial cell (magenta).
(I) Cross-sectional view of the VNC showing the same cells as in (H).
(J-K) Surface view (J) and oblique view (K) of the VNC showing two ventral channel subperineurial glia (cyan and magenta) with long extensions towards the neuropil.
(L) Lateral view of the VNC showing channel subperineurial glia on the ventral and dorsal surfaces, sending projections along the channels, and channel-only subperineurial glia, which reside along the channels only, both forming tube-like structures.
(M) Cross-sectional view of the VNC showing a single cortex glial cell in the cortical region forming a membranous, honeycomb-like structure.
(N-O) Lateral view (N) and cross-sectional view (O) of the VNC showing ensheathing glial cells at the border of the cortex and neuropil. Tract ensheathing cells extend longer or shorter processes through axon tracts entering the neuropil, while neuropil ensheathing cells only extend processes along the neuropil border.
(P) Cross-sectional view of the VNC showing a type 1 astrocyte and a type 2 astrocyte sending processes into the neuropil. Type 1 astrocyte processes were highly ramified, whereas type 2 astrocyte processes were less ramified. Co-expression of Pros and Repo indicated astrocyte identity (insets).
(Q-S) Cross-sectional views of the VNC showing single astrocytes in greyscale, from dorsal, lateral and ventral nucleus positions, with corresponding morphological type 1 or 2 indicated.
Green marks Repo, and cyan and magenta mark MCFO clones in all panels, except (P) with Pros in magenta.
Dashed lines outline the neuropil and full lines outline the VNC. Clones represent samples at 0 h after larval hatching. All scale bars represent 10 µm.

c. As for the comment that anti-Moody antibody is not available: there is a commercially available antibody against Mdr65 which gives an excellent and very specific staining as published by the Bainton lab. Moreover, even in the absence of an antibody, the combination of (moody-lexA, lexAop-GFP, CG5080-Gal4, UAS-tdTomato) should allow definition of whether the respective cells are identical or not.

- In the first revision, to show that CG5080-expressing glia are indeed located on the outermost glial layer (and are therefore perineurial glia), we generated CG5080-Gal4 MCFO clones in a *repo-lexA>LexAop-myr::tdTomato* background. This experiment is very similar to that now suggested by the reviewer (i.e. moody-lexA, lexAop-GFP, CG5080-Gal4, UAS-tdTomato). We presented these results in Figure 1- figure supplement S1 of the revised manuscript, which shows that the CG5080-Gal4 clones occupy the outermost glial surface as there is no *repo-lexA* driven *myr::tdTomato* expression on the outer-most surface that does not overlap with the CG5080-Gal4 MCFO clone. Similar analyses (visualizing overlap or exclusion of signal at the surface) have been used for the same purposes in Awasaki et al., 2008 (Figure 1, C and F). Therefore, together with the data described in the points above, we are confident that CG5080-Gal4 labels perineurial glia.

3. The reviewer takes issue with the statement: "the gene wrapper, which is a known cell type-specific marker of cortex glia (Coutinho-Budd et al., 2017)". Instead, Coutinho-Budd used a promoter fragment of the gene wrapper, which is specific for cortex glia. In Noordermeer et al. (1998) wrapper is described by protein expression as being midline specific in the embryo. BDGP in situ data show few lateral cells during embryogenesis but these cells are not classified as cortex glia.

- Several groups have reported that among *repo*+ glia *wrapper* expression is specific to cortex glia (Allen et al., 2020; Corrales et al., 2022; Konstantinides et al., 2018) using RNAseq to identify *wrapper*-expressing cells. This has been validated by use of the *R54H02*(*wrapper fragment)-Gal4* (Coutinho-Budd et al., 2017; Konstantinides et al., 2018). Given that mRNA and the R54H02-Gal4 have the same expression, this enhancer fragment does recapitulate endogenous expression in larval and adult stages.
  We have modified the text as follows, “For example, cells in cluster 1 strongly expressed the gene *wrapper*, which other groups have previously reported as a cortex glia-specific marker with *wrapper* expression validated using a Gal4 driven by an intronic fragment from the *wrapper* locus *(R54H02-Gal4)* (Allen et al., 2020; Corrales et al., 2022; Coutinho-Budd et al., 2017; Konstantinides et al., 2018).”
- As suggested by the reviewer’s previous comments, we highlighted *wrapper* expression in embryonic midline glia and these data were included in Figure 3- figure S1G of our previous revised submission.

4. In addition, this reviewer believes that validation of expression patterns using enhancer-Gal4 constructs is generaxlly problematic. CRIMIC or Trojan-Gal4 insertions should be used. please respond with specific arguments about using the constructs you used versus these insertions.

- We fully agree with Reviewer 1 and direct them to Supplementary File 4 of our manuscript, which provides the list of complete genotypes used by figure panel and which shows that most of the Gal4s we used to validate gene expression patterns in our manuscript are indeed CRIMIC-Gal4s, Trojan-Gal4s, or other enhancer trap lines (16 out of 21 lines). Note that the 5 lines that represent exogenously integrated enhancer or promoter Gal4 fusions are well-validated in previous literature: *repo* (Awasaki et al., 2008; Sepp et al., 2001; Stork et al., 2008), *alrm* (Doherty et al., 2009; Stork et al., 2014); *wrapper* (Bannerjee et al., 2017), *moody* (Bainton et al., 2005; Schwabe et al., 2005), *R54H02 (wrapper fragment)* (Coutinho-Budd et al., 2017; Konstantinides et al., 2018), and *R25H07* (Ackerman et al., 2021; Bajar et al., 2022; Hilu-Dadia et al., 2018).
- Please note that for figures related to the adult optic lobes, we validated gene expression using fluorescent *in situ* hybridization chain reaction (or with IHC and protein traps for a few

cases). We used well-characterised Janelia enhancer Gal4 lines in these experiments to label glial classes but not to draw conclusions on gene expression.

5. Pippin is reported to be expressed in perineurial and subperineurial glia (McMullen et al 2021); please cite this reference.

- We have included this citation on Page 13:
  "We note that similar to a previous report (McMullen et al., 2021), *pippin* is also expressed in subperineurial glia at low levels (Figure 4B), though we did not recover any subperineurial clones with the *pippin-Gal4* driver (Figure 4-figure supplement 1I; see below for further resolution of perineurial and subperineurial glial clusters)."

Lastly, Reviewer 3 thought that the word "detectable" in the title was not necessary, and we agree.

- Thank you, we have removed "detectable" from the title.

eLife decision: 01-08-2022-TR-eLife-82347R2

editorial@elifesciences.org <editorial@elifesciences.org>
Tue 08/08/2023 10:55
To:Fernandes, Vilaiwan <vilaiwan.fernandes@ucl.ac.uk>

⚠ Caution: External sender

Dear Dr Fernandes,

Thank you for resubmitting your work entitled "A *Drosophila* glial cell atlas reveals a mismatch between transcriptional diversity and morphological diversity" for further consideration by eLife. Your revised article has been evaluated by K VijayRaghavan (Senior Editor) and a Reviewing Editor.

The manuscript has been improved but there are some remaining issues that need to be addressed, as outlined in Reviewer 1's review below:

I believe that you have elucidated the morphological glial types in the VNC and examined their marker expression, for the most part addressing this reviewer's questions and concerns in lines 152-195 in the revised text of your manuscript. However, the text is a bit hard to follow because you don't always allude to marker expression for every group and the previously used nomenclature is difficult to match with the finer distinctions you make. A table of the glia you describe would be welcome, using your nomenclature, and indicating previously ascribed terms for the groups and subgroups, their morphology, and marker expression (moody, CG5080, etc). For example, on line 152, you refer to what had been called perineurial glia as surface-only perineurial glia with a fibrous morphology. You then allude to a "triple" subdivision (lines 193-195 "...subperineurial glia could be characterised roughly into three morphologies: the surface-only subperineurial glia, the channel-only subperineurial glia and those with intermediate morphologies".

In consultation with Reviewer 1 after submitting their re-review below, one additional amendment is to remove the "finding" of a new perineurial glial cell type. This reviewer argues that based on the Technau lab's data, the CG5080 marker overlaps with Moody which is a SPG marker. Reviewer 1 suggests using either a commercially available antibody against Mdr65 which as published by the Bainton lab gives excellent and specific staining. Or, in the absence of using an antibody, the combination of (moody-lexA, lexAop-GFP, CG5080-Gal4, UAS-tdTomato) should allow for resolving whether the respective cells are identical or not.

Reviewer #1 (Recommendations for the authors):

In the rebuttal letter the authors wrote:
Early studies did not distinguish between perineurial and sub-perineurial glia, instead Ito et al. (1995) state that: "The "surface-associated glia" category represents the cells that are closely associated with the CNS surface. The surface is covered by the perineurium, which consists of the acellular neural lamella (Scharrer 1939) and the surface-associated glial cells underneath (Fig. 3). In the embryonic and L1 VNC we defined two subtypes of this glial category. The "subperineurial glia" (SPG) lie beneath the outer surface of the VNC, and the

"channel glia" (CG) lie along the dorsoventral channels". Along similar lines, in 2008 Beckervordersandforth et al. state, "The category of surface-associated glia includes two subgroups: the subperineurial glia (SPG) that lie underneath the outer surface of the CNS, and the channel glia (CG) which are positioned along the dorsoventral channels". In other words, in these early reports "subperineurial glia" (SPG) include what the field now knows to be both the perineurial and subperineurial glial classes.

I disagree, the argument is circular. Today we do know that moody is an SPG marker, a very specific marker, indeed. If moody is expressed in the same cells as CG5080, then to me it is pretty clear that CG5080 is active in SPGs and not in PGs.

A clear demonstration that CG5080 is active in PGs is still missing. A solution would be an antibody staining, or the use of moody-LexA LexAop-tdTomato, CG5080-Gal4 UAS GFP. The data shown in Figure supplement 1 do not demonstrate that CG5080 positive cells are located at the outer surface of the CNS which would be expected for PGs.

We will look forward to hearing from you with a revised article and a response letter describing the changes made. We hope you will resubmit in a timely manner for further consideration and you can use the following link when you are ready to resubmit:

[https://submit.elifesciences.org/cgi-bin/main.plex?el=A5Hz5oth1C5BRJo3I2A9ftdPfFecXQK1XKlU7W1wToNgZ]

Best wishes,

Carol Mason
Reviewing Editor, eLife

K VijayRaghavan
Senior Editor, eLife

-----
eLife Sciences Publications, Ltd is a limited liability non-profit non-stock corporation incorporated in the State of Delaware, USA, with company number 5030732, and is registered in the UK with company number FC030576 and branch number BR015634 at the address Westbrook Centre, Milton Road, Cambridge, CB4 1YG, UK.

There were 2 main points raised in the most recent reviews:

(1) The reviewers felt that the text describing the embryonic VNC surface glial morphological categories was hard to follow. We took this feedback onboard and clarified the text in this section as detailed below. We also point the reviewers to Supplementary file 1, which provides a table (included in our previous revision) that places our morphological characterisation in the context of previous nomenclature.

(2) Reviewer 1 argues that *CG5080-Gal4* labels subperineurial glia and not perineurial glia. The premise of this argument is based on the reviewer's claim that Beckervordersandforth et al. (2008) demonstrated co-expression of *CG5080* and *moody* in subperineurial glia. As detailed in our previous response and again below, this is a misinterpretation of the results from Beckervordersandforth et al. (2008). Instead, there is no evidence to suggest that *CG5080* and *moody* are co-expressed in the same cells (see below and previous response)**.**

We use a combination of features including cell morphology, size, position, tiling properties, and marker expression to argue that *CG5080-Gal4* and *moody-Gal4* mark distinct surface glial classes, namely, perineurial glia and subperineurial glia respectively.

Please see our detailed response below in green.

I believe that you have elucidated the morphological glial types in the VNC and examined their marker expression, for the most part addressing this reviewer's questions and concerns in lines 152-195 in the revised text of your manuscript. However, the text is a bit hard to follow because you don't always allude to marker expression for every group and the previously used nomenclature is difficult to match with the finer distinctions you make. A table of the glia you describe would be welcome, using your nomenclature, and indicating previously ascribed terms for the groups and subgroups, their morphology, and marker expression (moody, CG5080, etc). For example, on line 152, you refer to what had been called perineurial glia as surface-only perineurial glia with a fibrous morphology. You then allude to a "triple" subdivision (lines 193-195 "...subperineurial glia could be characterised roughly into three morphologies: the surface-only subperineurial glia, the channel-only subperineurial glia and those with intermediate morphologies".

- We have taken this feedback onboard and clarified the text in this section, which has improved the readability of the manuscript (see details below). We also point the reviewers to Supplementary file 1 (included in our previous revision), which provides a table that places our morphological characterisation in the context of previous nomenclature. Finally, we have clarified the "triple" subdivision of subperineurial glia throughout the manuscript.

Lines 125-171 now read as follows:

VNC surface glia

The *Drosophila* CNS is bathed in circulating hemolymph, which contacts the VNC at its main surface and along dorsoventral channels that perforate the VNC along the midline between pairs of longitudinal connectives and neighbouring neuromeres (1,2). The surface glia comprise two glial classes, perineurial on the outer surface of the VNC and subperineurial glia positioned below perineurial glia (reviewed in Freeman, 2015 and Pogodalla et al., 2022). Early glial studies did not distinguish between these two classes and

reported expression of the markers *CG5080* and *moody* in surface-associated glia along the main VNC surface and associated with the dorsoventral channels, a result also confirmed by bulk RNA profiling of surface glia (2,5,6). Later, Moody was shown to be expressed in subperineurial glia exclusively (7,8), while *CG5080* expression was not further characterised.

To analyse surface glia morphology in the VNC, we used the *CG5080-Gal4* and *moody-Gal4* drivers to generate MCFO clones. *CG5080-Gal4* labelled cells with fibrous membranes and which tiled loosely to cover the main surface of the VNC, typical characteristics of perineurial glia described by others (reviewed in Hartenstein, 2011), (Figure 1A-F). Therefore, it is likely that *CG5080-Gal4* labelled cells that belong to the perineurial glial class. Interestingly, *CG5080-Gal4* labelled cells located along the midline of the dorsal and ventral surfaces of the VNC each sent a single tapered projection inward along the dorsoventral channels (Figure 1C,D,F and Figure 1 – figure supplement 1A-C; N=51 clones from N=13 brains). When viewed at the surface, these cells tiled with and were indistinguishable from the other *CG5080-Gal4* labelled surface-only cells (Figure 1B-F; N=140 clones from N=13 brains). Ventral midline cells projected further than their dorsal counterparts and were present along the entire anterior-posterior axis of the VNC, whereas cells that projected from the dorsal surface were observed with lower frequency towards more posterior positions of the VNC (Figure 1D and Figure 1 – figure supplement 1A). To further confirm that the channel-associated cells belong to the perineurial glial class, we used *CG5080-Gal4* to label individual cells while co-labelling all glia with *repo-LexA>LexAop-myr::tdTomato.* We observed that the channel-associated cells occupied the outermost glial surface of the VNC (Figure 1 – figure supplement 1B,C). Taken together with the fact that these cells tiled with surface-only perineurial glia, these data argue that they are a subclass of perineurial glia. Hereafter we refer to them as 'channel-associated perineurial glia'.

Since *moody* is a known marker of subperineurial glia (7,8), we next generated MCFO clones labelled with *moody-Gal4.* Although we recovered cells at the surface of the VNC and associated with the dorsoventral channels, these did not resemble clones labelled by *CG5080-Gal4* (Figure 1A-F). Instead, consistent with previous reports of subperineurial glia (reviewed in Hartenstein, 2011), *moody-Gal4* MCFO clones at the surface of the VNC were polygonal in shape, tiled tightly with each other, were larger than *CG5080-Gal4* labelled cells, and displayed a honeycomb-like pattern within their cell boundaries, as a result of their membranes cupping neuronal cell bodies at the outer surface of the cortex (10)(Figure 1A, G-L and Figure 1 – figure supplement 1D). While most cells labelled by *moody-Gal4* were only associated with the main surface of the VNC (69.3% of total clones were associated with the main surface only; N=101 clones from N=12 brains), some of the cells, present along the VNC midline sent short (non-tapering) projections along the dorsoventral channels (21.8% of total clones). In addition, *moody-Gal4* also labelled cells that exclusively lined the dorsoventral channels (8.9% of total clones; Figure 1H,I). Thus, we observed a continuum of glial morphologies between the two extremes of surface-only and channel-only subperineurial glia (Figure 1J-L). Importantly, glia with intermediate morphologies (*i.e.* which associated with both the surface and the channels), tiled tightly with neighbouring surface-only associated cells (Figure 1H,I).

Thus, based on morphology, tiling properties, size and position, these data argue that *moody-Gal4* labels the subperineurial glial class, which can be further subdivided into surface-only, surface- and channel-associated, and channel-only morphological categories. By contrast, *CG5080-Gal4* labels the perineurial glial class, which can be further subdivided into surface-only and channel-associated morphological categories.

In consultation with Reviewer 1 after submitting their re-review below, one additional amendment is to remove the "finding" of a new perineurial glial cell type. This reviewer argues that based on the Technau lab's data, the CG5080 marker overlaps with Moody which is a SPG marker.

Reviewer 1 suggests using either a commercially available antibody against Mdr65 which as published by the Bainton lab gives excellent and specific staining. Or, in the absence of using an antibody, the combination of (moody-lexA, lexAop-GFP, CG5080-Gal4, UAS-tdTomato) should allow for resolving whether the respective cells are identical or not.

Reviewer #1 (Recommendations for the authors):

In the rebuttal letter the authors wrote:
Early studies did not distinguish between perineurial and sub-perineurial glia, instead Ito et al. (1995) state that: "The "surface-associated glia" category represents the cells that are closely associated with the CNS surface. The surface is covered by the perineurium, which consists of the acellular neural lamella (Scharrer 1939) and the surface-associated glial cells underneath (Fig. 3). In the embryonic and L1 VNC we defined two subtypes of this glial category. The "subperineurial glia" (SPG) lie beneath the outer surface of the VNC, and the "channel glia" (CG) lie along the dorsoventral channels". Along similar lines, in 2008 Beckervordersandforth et al. state, "The category of surface-associated glia includes two subgroups: the subperineurial glia (SPG) that lie underneath the outer surface of the CNS, and the channel glia (CG) which are positioned along the dorsoventral channels". In other words, in these early reports "subperineurial glia" (SPG) include what the field now knows to be both the perineurial and subperineurial glial classes.

I disagree, the argument is circular. Today we do know that moody is an SPG marker, a very specific marker, indeed. If moody is expressed in the same cells as CG5080, then to me it is pretty clear that CG5080 is active in SPGs and not in PGs.

A clear demonstration that CG5080 is active in PGs is still missing. A solution would be an antibody staining, or the use of moody-LexA LexAop-tdTomato, CG5080-Gal4 UAS GFP. The data shown in Figure supplement 1 do not demonstrate that CG5080 positive cells are located at the outer surface of the CNS which would be expected for PGs.

The premise of the reviewer's argument is based on their claim that Beckervordersandforth et al. (2008) demonstrated co-expression of *CG5080* and *moody* in subperineurial glia*: "This reviewer argues that based on the Technau lab's data, the CG5080 marker overlaps with Moody"* and *"moody is expressed in the same cells as CG5080"*. As detailed in our previous rebuttal and again as follows, **this is a misinterpretation of the results from Beckervordersandforth et al. (2008).**

In Table 3 of Beckervordersandforth et al. (2008), *CG5080* and *moody* expression in **surface glia** were characterised by *in situ* hybridisation and evaluated as being expressed in "50-100% of hemisegments examined". The materials and methods section of their manuscript indicates that the authors performed single colour fluorescent *in situ* hybridisation, thus indicating that all *in situ* hybridisation experiments were performed individually as independent experiments, thus, **co-expression was not examined and, therefore, cannot and should not be inferred**. Furthermore, the authors stated whether a particular marker was expressed in either 50-100% or <50% of hemisegments examined,

i.e., in most instances, and especially for all instances where *in situ* hybridisation was used, **individual cells were not evaluated**. Thus, the reviewer's assertion that *CG5080* and *moody* are co-expressed is not valid.

We use a combination of well-established features to define the perineurial glial class. We show that clones labelled by *CG5080-Gal4* possessed morphological features ascribed to perineurial glia (reviewed in Hartenstein, 2011): they loosely tiled with each other irrespective of whether they possessed projections associated with channels or were only surface-associated (note that this point was discussed and agreed upon as an essential revision by all three reviewers) and their surface domains were characterised by fibrous membranes. Importantly, these cells were morphologically distinct when compared to clones labelled by *moody-Gal4* (a well-established subperineurial glia, as pointed out by reviewer 1). *moody>MCFO clones* were polygonal in shape, tiled tightly with each other, possessed "many ring-like membranous structures within the cells […which are] cap-like basal protrusions that cover individual neuronal cell bodies within the cortex", consistent with previous reports of subperineurial glia (Kremer et al., 2017; reviewed in Hartenstein, 2011). Moreover, *moody-Ga*l4 labelled cells were considerably larger than cells labelled by *CG5080-Gal4* (Fig. 1 A-L), another well-known feature of subperineurial versus perineurial glia.

We now include quantifications of the area at the main surface of the VNC occupied by the two perineurial glial morphological categories: surface-only and channel-associated, and the surface-only subperineurial glia as a new panel **(D)** in Figure 1- figure supplement S1.

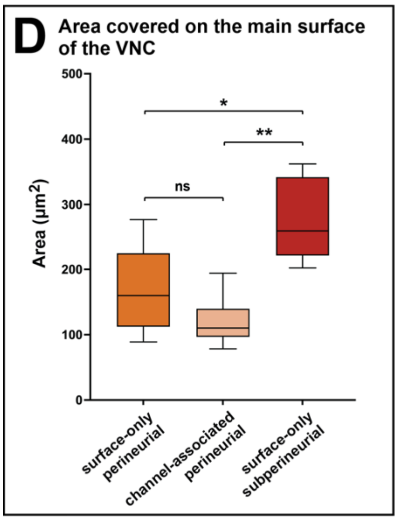

Lastly, we generated *CG5080-Gal4 MCFO* clones in a *repo-lexA>LexAop-myr::tdTomato* background. This experiment is very similar to the experiment suggested by the reviewer (i.e. *moody-lexA, lexAop-GFP, CG5080-Gal4, UAS-tdTomato*). We presented these results in Figure 1- figure supplement S1 of the revised manuscript, which shows that the *CG5080-Gal4* clones occupy the outermost glial surface as there is no *repo-lexA* driven *myr::tdTomato* expression on the outer-most surface that does not overlap with the *CG5080-Gal4 MCFO* clone.

Given these observations, as well as descriptions of perineurial versus subperineurial glia from the literature (Awasaki et al., 2008; Stork et al., 2008; Kremer et al., 2017) we are confident in our assertion that the *CG5080-Gal4* clones presented in Figure 1B-F correspond to perineurial and not subperineurial glia.
