# Supplementary material for: A Drosophila glial cell atlas reveals a mismatch between transcriptional and morphological diversity
Source: PLoS Biol. 2023 Oct 20;21(10):e3002328. doi: 10.1371/journal.pbio.3002328 (PMC10619882; doi:10.1371/journal.pbio.3002328)
Supplement: Attachment — Submitted filename: Response to reviews.pdf [file pbio.3002328.s043.pdf]

Thank you for your feedback. We are pleased that our study was seen to be suitable for publication at PLOS Biology with minor revisions. As part of these revisions, we were asked to clarify the perceived discrepancy of whether *CG5080* is a perineurial vs subperineurial marker. We have addressed this along with all other editorial requests as detailed below in green. This has improved the manuscript and helps contextualize our data.

From the review history, we see that reviewers 2 and 3 from eLife were satisfied by the revision but reviewer 1 had some lingering concerns about the specificity of CG5080-Gal4 as a marker of perineurial glia. We see that that reviewer 1 argued that findings from an earlier report (Beckervordersandforth et al., 2008) suggests that CG5080 is coexpressed with the SPG marker moody - and reviewer 1 felt that more work was needed to demonstrate CG5080 as a marker of PGs.

We understand that you disagree with this interpretation, noting that CG5080 and moody co-expression was actually not directly shown in the previous work. Our Academic Editor largely agrees with your interpretation of the previous study, and s/he thinks the the current data provided is adequate to support your conclusions about perineurial glial cell types. Therefore, based on this assessment, we would not require that this conclusion be removed or experimentally strengthened before publication.

However, as the disagreement comes down to interpretation, and as other readers may have similar questions as reviewer 1, we suggest that you consider adding further discussion regarding reviewer 1's comments to the manuscript. We think it would be helpful to outline in a few sentences detailing why there is a perceived discrepancy. In particular, the the Academic Editor suggests you could expand on this comment from the results "Thus, based on morphology, tiling properties, size and position, these data argue that moody-Gal4 labels the subperineurial glial class, which can be further subdivided into surface-only, surface- and channel-associated, and channel-only morphological categories. By contrast, CG5080- Gal4 labels the perineurial glial class, which can be further subdivided into surface-only and channel associated morphological categories."

To clarify for the reader any perceived discrepancy of whether *CG5080* is a perineurial vs subperineurial marker we have expanded the text in two parts. First, we added to the introduction of the VNC surface glia results section to provide more detail and context for early glial studies, and second, we expanded our closing paragraph of the results section, as suggested by the academic editor. These changes are highlighted in our revised manuscript and pasted below.

Lines 120-185, changes highlighted:

The *Drosophila* CNS is bathed in circulating hemolymph, which contacts the VNC at its main surface and along dorsoventral channels that perforate the VNC along the midline between pairs of longitudinal connectives and neighbouring neuromeres (43,44). The surface glia comprise two glial classes, perineurial on the outer surface of the VNC and subperineurial glia positioned below perineurial glia (reviewed in Freeman, 2015 and Pogodalla et al., 2022). Early glial studies did not distinguish between these two classes and referred to both collectively as subperineurial glia for their position below the perineurium (44,45). An early characterization of gene expression in glia of the embryo reported *CG5080* and *moody* expression in surface glia along the main VNC surface and associated with the dorsoventral channels ('channel glia'), a result since confirmed by bulk RNA profiling of surface glia (45,46). It is important to note that these studies did not evaluate glial morphology, whether *CG5080* and *moody* were expressed by both surface glial classes nor whether they were co-expressed in the same cells (45,46). Indeed, Moody has since been shown to be expressed exclusively in subperineurial glia (47,48). However, *CG5080* expression has not been characterised further, thus implying three possibilities: (1) it may be expressed in both perineurial and

sub-perineurial glia, (2) it may be expressed in perineurial glia exclusively or (3) it may be expressed in sub-perineurial glia exclusively.

To analyse surface glia morphology in the VNC, we used the *CG5080-Gal4* and *moody-Gal4* drivers to generate MCFO clones. *CG5080-Gal4* labelled cells with fibrous membranes and which tiled loosely with each other to cover the main surface of the VNC, typical characteristics of perineurial glia described by others (38), (Figure 1A-F). Therefore, it is likely that *CG5080-Gal4* labelled cells that belong to the perineurial glial class. Interestingly, *CG5080-Gal4* labelled cells located along the midline of the dorsal and ventral surfaces of the VNC each sent a single tapered projection inward along the dorsoventral channels (Figure 1C,D,F and Figure 1 – figure supplement 1A-C; N=51 clones from N=13 brains). When viewed at the surface, these cells tiled with and were indistinguishable from the other *CG5080-Gal4* labelled surface-only cells (Figure 1B-F; N=140 clones from N=13 brains). Ventral midline cells projected further than their dorsal counterparts and were present along the entire anterior-posterior axis of the VNC, whereas cells that projected from the dorsal surface were observed with lower frequency towards more posterior positions of the VNC (Figure 1D and Figure 1 – figure supplement 1A). To further confirm that the channel-associated cells belong to the perineurial glial class, we used *CG5080-Gal4* to label individual cells while co-labelling all glia with *repo-LexA>LexAop-myr::tdTomato.* We observed that the channel-associated cells occupied the outermost glial surface of the VNC (Figure 1 – figure supplement 1B,C). Taken together with the fact that these cells tiled with surface-only perineurial glia, these data argue that they are a subclass of perineurial glia. Hereafter we refer to them as ‘channel-associated perineurial glia’.

Since *moody* is a known marker of subperineurial glia (47,48), we next generated MCFO clones labelled with *moody-Gal4.* Although we recovered cells at the surface of the VNC and associated with the dorsoventral channels, these did not resemble clones labelled by *CG5080-Gal4* (Figure 1A-F). Instead, consistent with previous reports of subperineurial glia (38), *moody-Gal4* MCFO clones at the surface of the VNC were polygonal in shape, tiled tightly with each other, were larger than *CG5080-Gal4* labelled cells, and displayed a honeycomb-like pattern within their cell boundaries, as a result of their membranes cupping neuronal cell bodies at the outer surface of the cortex (25)(Figure 1A, G-L and Figure 1 – figure supplement 1D). While most cells labelled by *moody-Gal4* were only associated with the main surface of the VNC (69.3% of total clones were associated with the main surface only; N=101 clones from N=12 brains), some of the cells, present along the VNC midline sent short (non-tapering) projections along the dorsoventral channels (21.8% of total clones). In addition, *moody-Gal4* also labelled cells that exclusively lined the dorsoventral channels (8.9% of total clones; Figure 1H,I). Thus, we observed a continuum of glial morphologies between the two extremes of surface-only and channel-only subperineurial glia (Figure 1J-L). Importantly, glia with intermediate morphologies (*i.e.* which associated with both the surface and the channels), tiled tightly with neighbouring surface-only associated cells (Figure 1H,I).

As aforementioned, *CG5080* and *moody* were reported to be expressed in surface glia though co-expression and expression across both surface glial classes were not assessed (45,46). Here we used *CG5080-Gal4* and *moody-Gal4*-labelled MCFO clones to evaluate cell morphology, tiling properties, size and position, and clarify whether these labelled the same or different surface glial classes. Consistent with previous reports (38,47,48), our data argue that *moody-Gal4* labels the subperineurial glial class, which can be further subdivided into surface-only, surface- and channel-associated, and channel-only morphological categories. By contrast, our data suggest that *CG5080-Gal4* labels the perineurial glial class, which can be further subdivided into surface-only and channel-associated morphological categories. *CG5080-Gal4* labelled cells did not resemble cells labelled by *moody-Gal4*. Instead, they covered smaller domains at the outermost surface of the brain, possessed fibrous membranes and tiled loosely with each other; all features that are associated with perineurial glia (38). Thus, we confirm *moody* as a marker of subperineurial glia, and identify CG5080 as a new marker of perineurial glia. Moreover, we show that surface glia associated with the dorsoventral channels (referred to as ‘channel glia’ in early reports) (44,45) also consist of perineurial and subperineurial glia, which had not been appreciated previously.

1) TITLE: We would like to suggest a minor tweak to streamline the title. If you agree, we suggest it be changed to: "A Drosophila glial cell atlas reveals a mismatch between transcriptional and morphological diversity"
We have amended the title as requested.

2) DATA AND CODE:
a. Please update the github link https://github.com/AustinSeroka/2022_stage17_glia, to include a readme file, detailing what the various codes are and where they were applied in the manuscript.
We have included a readme file for the embryo glia GitHub, which details what the various codes are and where they were applied in the manuscript.

b. Please add a sentence to every figure legend (including supplemental) indicating where the underling data can be found. For example, you can add the sentence "the data underlying this figure can be found at ___" (and then reference the relevant repository/supplemental file)
We have indicated in the figure legends in which repositories or supplemental files the underling data can be found.

3) DATA NOT SHOWN: Please note that per journal policy, we do not allow the mention of "data not shown", "personal communication", "manuscript in preparation" or other references to data that is not publicly available or contained within this manuscript.   I detected two instances of this on line 262 and 273. Please either remove mention of these data or provide figures presenting the results and the data underlying the figure(s).
We have removed all instances of 'data not shown' from the manuscript.

As you address these items, please take this last chance to review your reference list to ensure that it is complete and correct. If you have cited papers that have been retracted, please include the rationale for doing so in the manuscript text, or remove these references and replace them with relevant current references. Any changes to the reference list should be mentioned in the cover letter that accompanies your revised manuscript.
We have not made any changes to the reference list.
